# Assessment of Small Mammal Communities in Forested Areas of the Sintra-Cascais Natural Park (Portugal)

**DOI:** 10.3390/ani16162590

**Published:** 2026-08-19

**Authors:** Joaquim T. Tapisso, Rita I. Monarca, Ana M. Cerveira, Sophie von Merten, Maílis Carrilho, Guilherme Aparício, Maria da Graça Ramalhinho, Maria da Luz Mathias

**Affiliations:** 1 Centro de Ecologia, Evolução e Alterações Ambientais (Ce3C), Global Change and Sustainability Institute, Faculdade de Ciências da Universidade de Lisboa, Campo Grande, 1749-016 Lisboa, Portugalapcerveira@ciencias.ulisboa.pt (A.M.C.);; 2 Department of Environment and Biodiversity, University of Salzburg, Hellbrunner Straße 34, 5020 Salzburg, Austria; 3 Museu Nacional de História Natural e Ciência, Rua da Escola Politécnica 58, 1250-100 Lisboa, Portugal

**Keywords:** small mammals, trapping survey, forests, management, conservation

## Abstract

For this project, we surveyed small mammal communities in protected forest habitats of the Sintra-Cascais Natural Park, Portugal, to establish a robust baseline for conservation planning. Our study provides a dataset and accompanying information to support an empirical framework for assessing the environmental determinants of small mammal occurrence across diverse habitats. These habitats encompass the park’s main forested areas within the UNESCO-designated landscape of the Sintra Mountain Range. Field sampling was conducted in two periods (2018 and 2024–2025) across six areas, comprising a total of 43 sites representative of the dominant habitat types within each location. A combination of live trapping, hair sampling, and camera trapping was employed, which enabled the detection of ten small mammal species, four Eulipotyphla and six Rodentia. The final dataset, derived from an extensive sampling effort of 8820 trap-nights, comprises a total of 792 specimen records. The complementary sampling approaches contributed differently to species detection, with live trapping recording the highest species richness and camera trapping generating the greatest number of detections. Obtained data provide critical support to inform the design and implementation of integrated, evidence-based conservation actions to sustain stable small mammal populations over the long term.

## Simple Summary

Protected areas, including natural parks, are central to biodiversity conservation through habitat protection and the adaptive monitoring and management of species and ecosystems. The Sintra-Cascais Natural Park, located in Portugal, was established in response to growing urban development and tourism pressures that posed significant threats to an area of high environmental sensitivity. Rich in natural values, the park is dominated by the distinctive landscape of the Sintra Mountain Range, a UNESCO World Heritage Site. It features a mosaic of oak, cypress, pine, acacia, and eucalyptus forests, interspersed with Mediterranean shrublands, collectively supporting a diverse and unique biota. This study aims to improve understanding of the composition of small mammal communities across representative forest stands, focusing on the relative abundance of each species, their spatial distribution, and key occupied habitats. The results will serve as a foundation for the development of policies and implementation of future integrated conservation strategies.

## 1. Introduction

Protected areas play a crucial role in biodiversity conservation by safeguarding ecosystems, offering refuge for threatened species, and functioning as reference areas for long-term ecological monitoring [1,2]. In addition, they also serve as relevant infrastructures for advancing scientific research, fostering environmental education, and supporting recreation under sustainable management frameworks. However, the effectiveness of these actions largely depends on the implementation of adaptive management strategies, which must be continuously revised and refined in response to emerging scientific evidence and dynamic environmental conditions [3,4,5]. Under this context the present study provides a field-based assessment of the small mammal communities occurring in the forestry areas of the Sintra-Cascais Natural Park (PNSC, Portugal).

The PNSC was established in 1994 [6] and spans approximately 14,580 ha along Portugal’s central-western coast. The park’s topography is defined by the Sintra Mountain Range, a distinctive landscape that has been designated a UNESCO World Heritage Site since 1995 due to its exceptional natural and cultural values [7].

The park reaches a maximum elevation of 528 m and experiences a temperate Mediterranean climate with pronounced Atlantic influence, characterized by high humidity levels resulting from the role of the Sintra Mountain Range as a condensation barrier. The combination of diverse geological formations and climatic diversity supports a wide array of habitats, dominated by oak, cypress, acacia, and eucalyptus forests, pinewoods, and shrublands [8]. According to the IUCN system [9] the PNSC is designated as a Category V protected area, assigned to areas of significant ecological, biological, cultural, or scenic value. Such areas are managed to ensure the coexistence of human activities and natural processes, thereby supporting sustainable practices, including traditional agriculture and forestry. These principles underpin the current strategy implemented by Parques de Sintra, Monte da Lua S.A. (PSML), the authority responsible for the management of the park’s forestry areas.

Here, we describe the sampling methodologies, data collection procedures, and analytical approaches employed to assess small mammal biodiversity within habitats managed by PSML, with particular emphasis on elucidating species-habitat associations and how these ultimately shape species distributions.

Specifically, the study aimed to (i) document the small mammal communities, (ii) determine their relative abundance and frequency; and (iii) evaluate species distribution across the primary habitats within the study area.

By addressing these objectives, the study also aims to generate scientific data to inform a dynamic, adaptive management framework, essential for maintaining the integrity of forestry ecosystems in the PNSC and ensuring stable small mammal populations. This study highlights the ecological and cultural significance of the Sintra Mountain Range, providing a scientific basis for evidence-based conservation strategies.

## 2. Materials and Methods

### 2.1. Study Area Description

The study primarily focused on the forested areas of the Sintra-Cascais Natural Park (PNSC), located in the westernmost region of Portugal (38.77755, −9.41980), about 25 km from Lisbon, the country’s capital.

**Figure 1:**
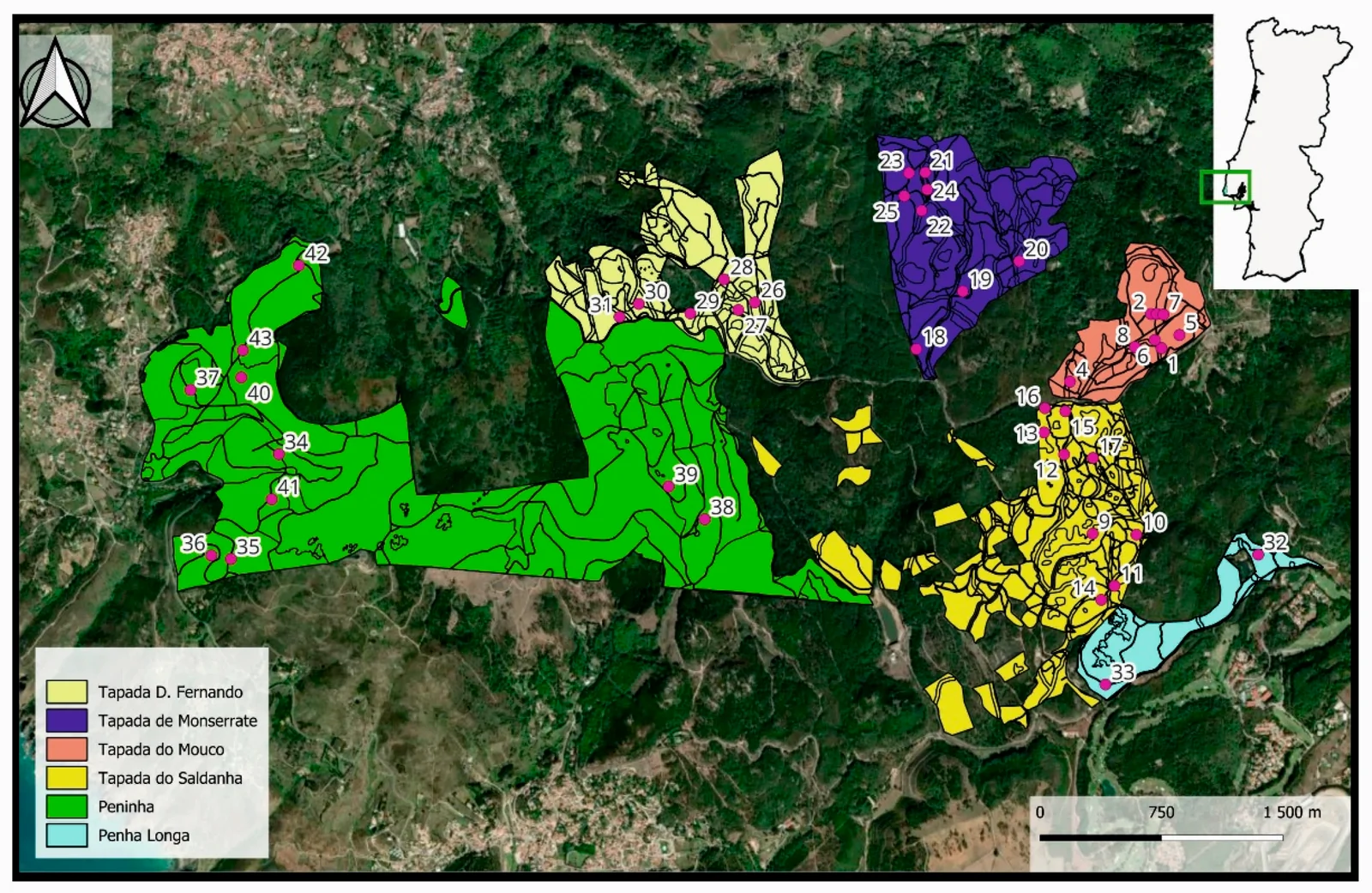
Forest stands and sites (1–43). Additional information is provided in Table 1 and Supplementary Materials S1 and S2.

Approximately one third of the main wooded core area of the Sintra Mountain is included in the PNSC, an area expected to support a diverse assemblage of small mammal species, many of which remain insufficiently documented. To address this knowledge gap, we selected six forest stands covering a total area of approximately 800 ha, representative of the dominant forest communities within the park, as previously identified in the forest management plan of the park (Plano de Gestão Florestal Parques de Sintra) [10]. The forest stands were identified from existing shapefiles prior to fieldwork, which guided the final selection of the 43 sampling sites. Between two and ten sites were selected per stand to capture the diversity of habitat patches within the study area (Figure 1, Table 1).

The six selected forest stands, here designated as stands A to F, were surveyed during two study periods (February to November 2018 and November 2024 to April 2025) across the 43 sites, sequentially numbered (1–43) and identified by the coordinates of their centroids (Table 1). The distance between sites in each stand ranged from 0.1 km to 6 km.

**Figure 2:**
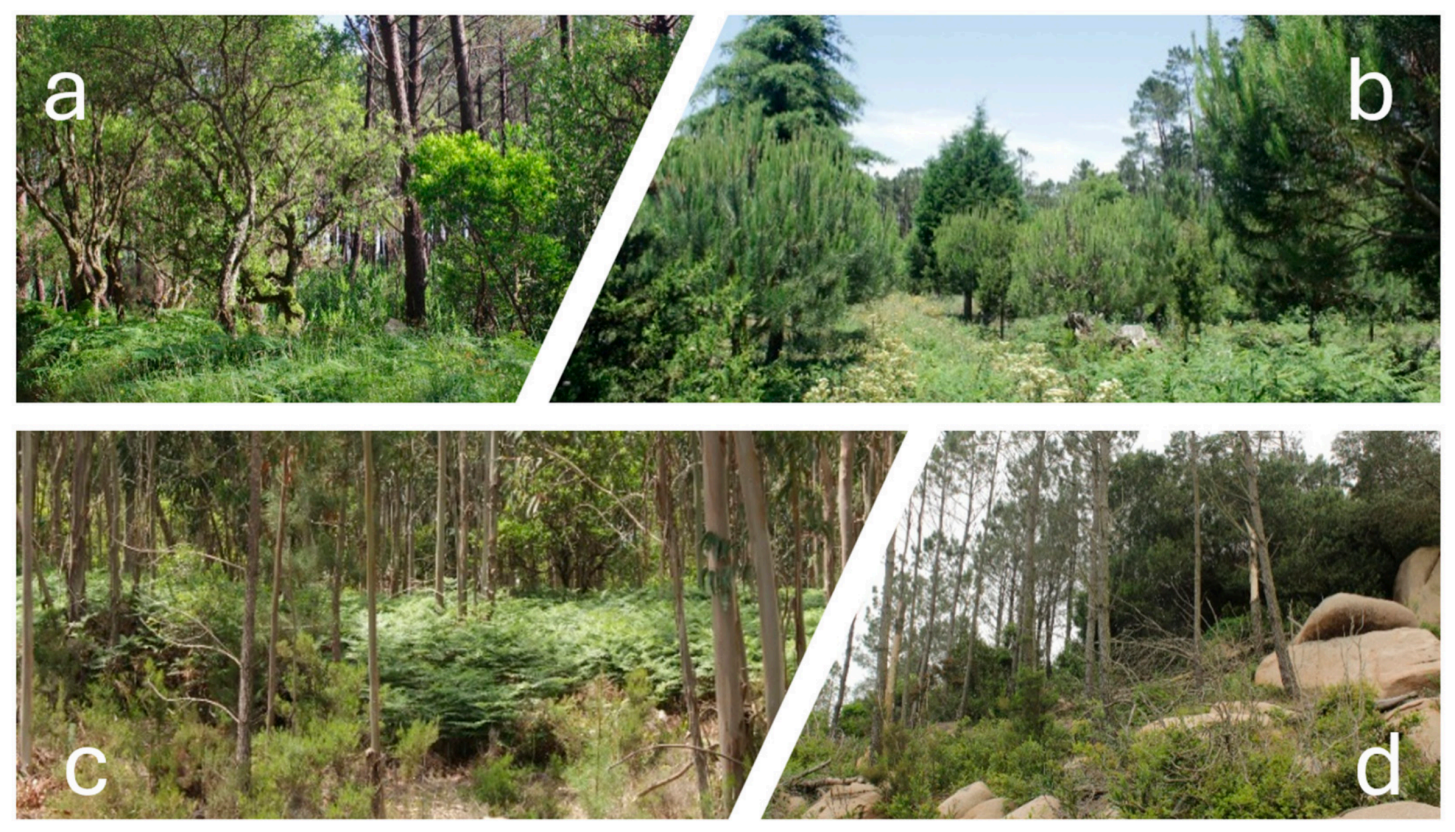
Representative forest communities within the Sintra-Cascais Natural Park: (**a**) oak mixed forest (site 18), (**b**) cypress mixed forest (site 26), (**c**) eucalyptus plantation (site 10), (**d**) pinewood (site 14).

Site selection accounted for factors such as dominant forest communities, understory composition, stand size, and accessibility to ensure efficient data collection (Figure 2).

### 2.2. Species Sampling

**Figure 3:**
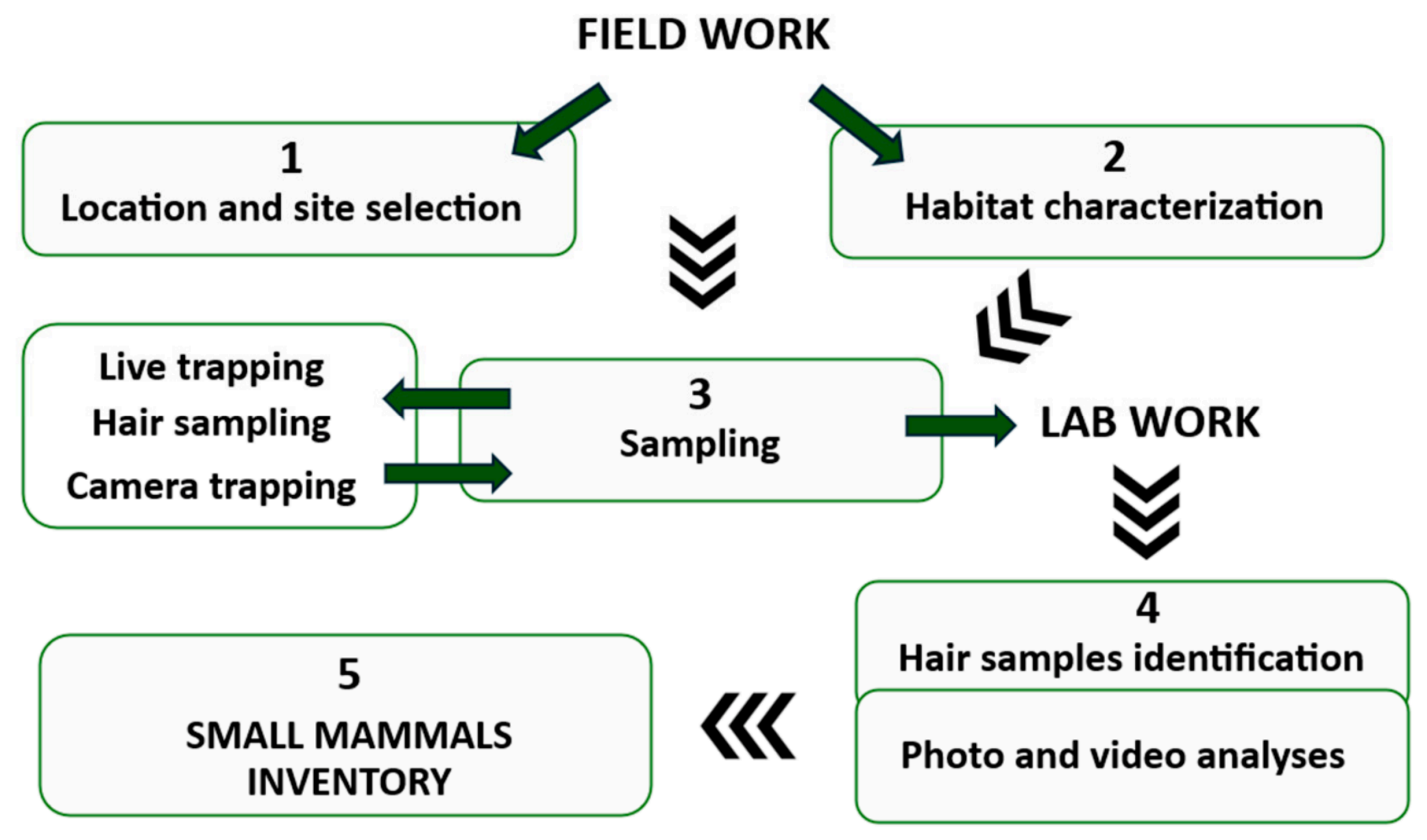
Flowchart outlining the methodological steps.

The small mammal inventory was carried out using a combination of live trapping, hair sampling, and camera trapping, designed to capture data on species with diverse ecological traits and habitat preferences (Figure 3). Live trapping was mainly used to ensure accurate species identification [11,12,13]. Non-invasive hair sampling was selected for its proven effectiveness in monitoring small mammal diversity. This method facilitates the detection of low-abundance species that would otherwise require significantly greater effort using live trapping alone, while also minimizing animal stress and disturbance [14,15,16]. Camera trapping was chosen for its documented success in detecting small mammals in forested and structurally complex landscapes, while also providing a cost-efficient survey method with reduced impacts on animal welfare [17,18]. These sampling methods were applied in different locations and sampling periods (2018 and 2024–2025), and therefore differences in sampling method, location, and timing may have influenced species detection and the resulting inventory.

During the first study period (February to November 2018), 31 sites across four forest stands were sampled and only live trapping and hair sampling were employed, with particular emphasis being placed on hair sampling. The remaining 12 sites, distributed across two forest stands, were surveyed during the second period (November 2024 to April 2025), and due to logistical constraints, only camera trapping was employed.

As the three methods were implemented in different locations (Table 1), during distinct time periods, the resulting datasets, although based on similar procedures, were not interpreted on a comparative basis; rather, they were considered complementary sources of evidence contributing to the attainment of the study’s main objectives.

The retrieved records were identified based on hair morphology (for details, see subsection Hair sampling) or morphological characteristics (for details, see subsections Live trapping and Camera trapping), including body size, body shape, and color patterns following the taxonomic classification in Wilson and Mittermeier [19] and Wilson et al. [20,21], and validated in accordance with the Mammal Diversity Database by the American Society of Mammalogists [22]. Species common names were aligned with those used by the previously cited authors, and their threatened status was assessed according to the Red Data Book of Mammals in Mainland Portugal [23]. Information on morphological characteristics, age, ecology, and habitat preferences for each species was obtained from the literature (e.g., [23,24]). All team members assisted in validating records up to species level whenever there was uncertainty in the taxonomic identification based on hair samples or video analyses; however, when it was impossible to obtain a high-confidence taxonomic identification up to species or genus levels, which was the case for two mouse species, these records were classified as ‘mice’ (e.g., [17,18]).

### 2.3. Sampling Description

**Figure 4:**
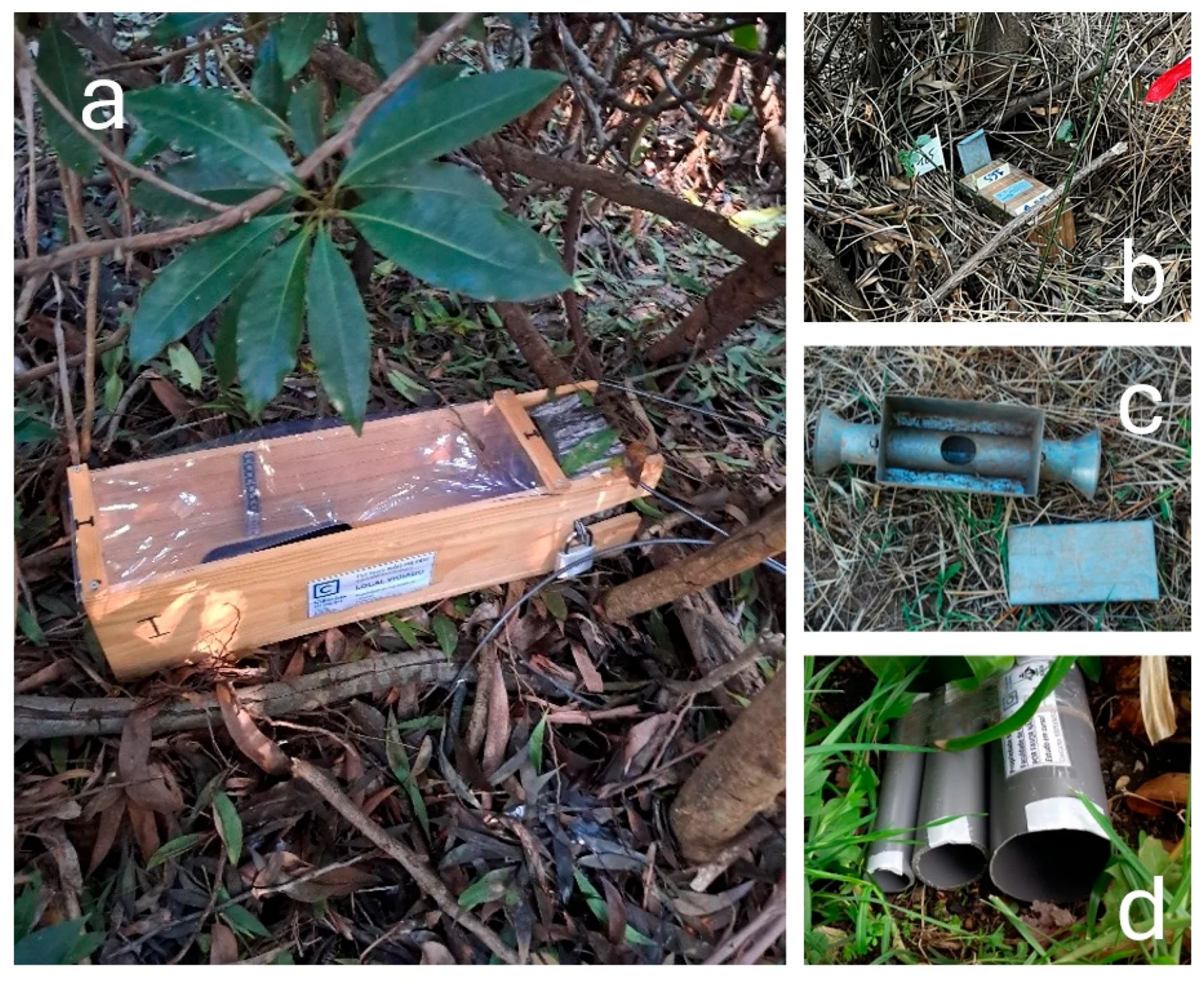
Trap models used in the species inventory: (**a**) camera trap, (**b**) wooden box live trap, (**c**) mole trap, (**d**) hair tubes.

Live trapping: Small mammals were live-captured using wooden box live traps (16.5 × 8.0 × 9.5 cm; PPUH A. Marcinkiewicz, Rajgród, Poland) baited with minced meat (Figure 4). Traps were set shortly before sunset and checked two to three times per night at two-hour intervals. Trapping was conducted across four forest stands (in total in 17 sites; see Table 1). In each stand, 120 traps were deployed for three consecutive nights, with trapping effort (number of traps × number of nights) varying between 60 and 180 trap-nights per site, totaling 360 trap-nights per stand. At one site (site 3), where signs of fossorial species were observed, an additional 120 mole traps were set and checked periodically over five days, contributing an extra 600 trap-nights to the total effective trapping effort of 2040 trap-nights across the four forest stands.

At the time of capture, small mammals were identified to species level and assigned to an age class (juvenile or adult) based on body weight and pelage coloration. Individuals were also fur-marked (small patch of fur cut from the dorsal abdominal region with a small scissor) for future recapture identification and released at the point of capture.

For live trapping data processing, the following analyses were conducted (e.g., [18,25,26]):

- Detection rate: The number of captures per 100 trap-nights, also used as a proxy of relative abundance, was calculated as the number of independent captures per species per site, multiplied by 100, and divided by the total number of trap-nights at that site, corresponding directly to the number of individuals of each species captured (excluding recaptures).
- Trap time-effort: The number of trap-nights required to obtain an independent species detection was calculated by dividing the number of trap-nights by the number of independent detections for that species.
- Inventory completeness rate: Defined as the number of species recorded by live trapping relative to the species richness estimated from the combined results of all three methods used.

Some individuals were captured multiple times during a session, indicating the presence of stable populations. A detailed account of the species recorded through live trapping is provided in the Supplementary Materials S1–S3.

Hair sampling: In addition to the live trapping survey, hair sampling was conducted at 24 of the 31 sites selected in the first campaign, with six sites sampled in each of the four stands (Table 1). Hair traps consisted of sets of three juxtaposed PVC tubes, with diameters of 32, 50, and 90 mm and lengths ranging from 12 to 15 cm. The use of different tube diameters accommodated species of varying sizes and enhanced trap stability in the field (Figure 4). At each sampling site, 30 hair-tube sets were installed, spaced 10 m apart, allowing for hair collection over a linear distance of 300 m per site. Hair samples were collected on small strips of double-sided adhesive tape placed inside the inner top of the tubes, one strip per tube. To maximize the likelihood of small mammals entering the tubes and depositing hair, tubes were baited with minced meat and left in the field for seven days, resulting in a trapping effort of 210 trap-nights per site, and 5040 trap-nights in total. The adhesive strips were retrieved from each tube and stored for subsequent analysis.

For species identification, collected hairs were prepared and mounted on microscope slides for the analysis of cuticular patterns, medullae structures and cross-section shapes of each sample. Cuticular imprints were made with transparent nail polish directly on the slides. Medullae were visualized by treating hairs with hydrogen peroxide prior to mounting, following the published literature [27,28,29]. For cross-sections, individual strands of hair were embedded in transparent nail polish, dried and then cut with a razor blade and mounted on the microscope slide. Hair samples were analyzed microscopically and identified to species level based on the abovementioned characteristics using diagnostic keys [27,29] and validated against a reference collection maintained by the research team to ensure accurate species-level identification.

Each set of three tubes was treated as a single sampling unit for calculating species presence/absence. Traps could contain hair from multiple species or multiple hairs from a single species. As it was not possible to determine if multiple hair samples of the same species came from one or multiple individuals, only one presence per species per trap was recorded.

Data assessment procedures followed those previously described for live trapping, with the difference that detections rates were based not on the number of animals captured, but on the number of presences of each species recorded per sampling unit.

Camera trapping: Camera-trap surveys were conducted at a total of 12 sites, deploying a single camera per site (Bushnell Trophy Cam HDE3, Minnesota, USA, with an infrared sensor LED Glow 32 Night Vision Flash to detect animal movement), over the six-month period of the second sampling campaign. This represented a trapping effort of 90 to 180 trap-nights per site, and a total of 1740 trap-nights. The cameras were placed inside wooden boxes (60 cm long, 16 cm wide, and 16 cm high; Figure 4) and equipped with close-focus lenses (+3 or +4 dioptres), allowing the focal plane to be adjusted to 10–40 cm. This setup improves the detection of small-bodied animals, particularly small mammals, and reduces the number of accidental images, such as those of people walking by. The camera-trap boxes were placed on the ground, baited with a pricked sardine can, and were periodically checked to ensure proper functioning. Bait, batteries and memory cards were replaced monthly.

Cameras were installed beneath vegetation or rocks in locations likely to provide cover and protection for small mammals, and they operated 24 h a day, recording the time and date of each visit. Cameras were set to record 15 s videos, with 60 s lapses to reduce multiple detection of the same individuals.

Data assessment procedures followed those previously described for live trapping, except that we considered the total number of independent recordings of each species over the survey period, rather than the number of individuals (live trapping) or the number of presences (hair sampling) (e.g., [18,30]) (Supplementary Materials S1–S3).

All detections were identified to species level (except for some individuals of two mouse species, as mentioned above) and assigned precise timestamps (date and time). We defined only the first recording of a species at a site each day as an independent detection, although consecutive detections of the same species at the same site could occur. Thus, any occurrence of a given taxon or species within a 24 h period was recorded as ‘one’ (see [17]), even if multiple individuals of the same taxon or species appeared within the same daily recording sequence. While this approach is considered to provide high level of confidence in the validity of detection events [17], it cannot prevent the repeated recording of the same individuals on consecutive days.

### 2.4. Diversity Indices

Species diversity was assessed using the Shannon diversity index (*H′*) and Pielou’s evenness index (*J′*). Shannon diversity was calculated as

$$H^{\prime }=-\sum_{i=1}^{S}p_{i}\ln p_{i}$$

where *p_i_* is the relative abundance of species *i* and *S* is the species richness. Pielou’s evenness was calculated as

$$J^{\prime }=H^{\prime }/\ln S$$

where *S* represents species richness. Both indices were calculated for each sampled forest stand based on the species abundances recorded at each location.

## 3. Results

### 3.1. Small Mammal Assessment

A total field effort of 8820 trap-nights and the collection of 792 specimen records provided the basis for the identification of ten species/genera: four Eulipotyphla—the West European Hedgehog, *Erinaceus europaeus* (family Erinaceidae), the Greater White-toothed Shrew *Crocidura russula*, the Iberian Shrew *Sorex granarius* (family Soricidae), and the Iberian Mole *Talpa occidentalis* (family Talpidae); and six Rodentia—the European Garden Dormouse *Eliomys quercinus* (family Gliridae), the Wood Mouse *Apodemus sylvaticus*, the Algerian Mouse *Mus spretus*, the Lusitanian Pine Vole *Microtus lusitanicus* (family Cricetidae), the Eurasian Red Squirrel *Sciurus vulgaris* (family Sciuridae), unidentified rat *Rattus* sp. (family Muridae) and unidentified mice *A. sylvaticus* + *M. spretus* (‘mice’), (Table 2).

The majority of occurring species are classified as non-threatened at both global and national scales [23]. Notable exceptions include the Iberian shrew (*S. granarius*) and the garden dormouse (*E. quercinus*), whose conservation statuses in Portugal have been recently revised to Near Threatened (NT) and Vulnerable (VU), respectively [31,32].

The highest values of species richness were recorded in Tapada do Mouco and Tapada de Monserrate (nine and eight species, respectively), whereas Tapada D. Fernando and Penha Longa exhibited the lowest richness (four species in each location) (Table 3). The highest Shannon diversity and Pielou’s evenness were recorded in Tapada do Mouco, whereas Penha Longa exhibited the lowest values for both indices (Table 3).

**Figure 5:**
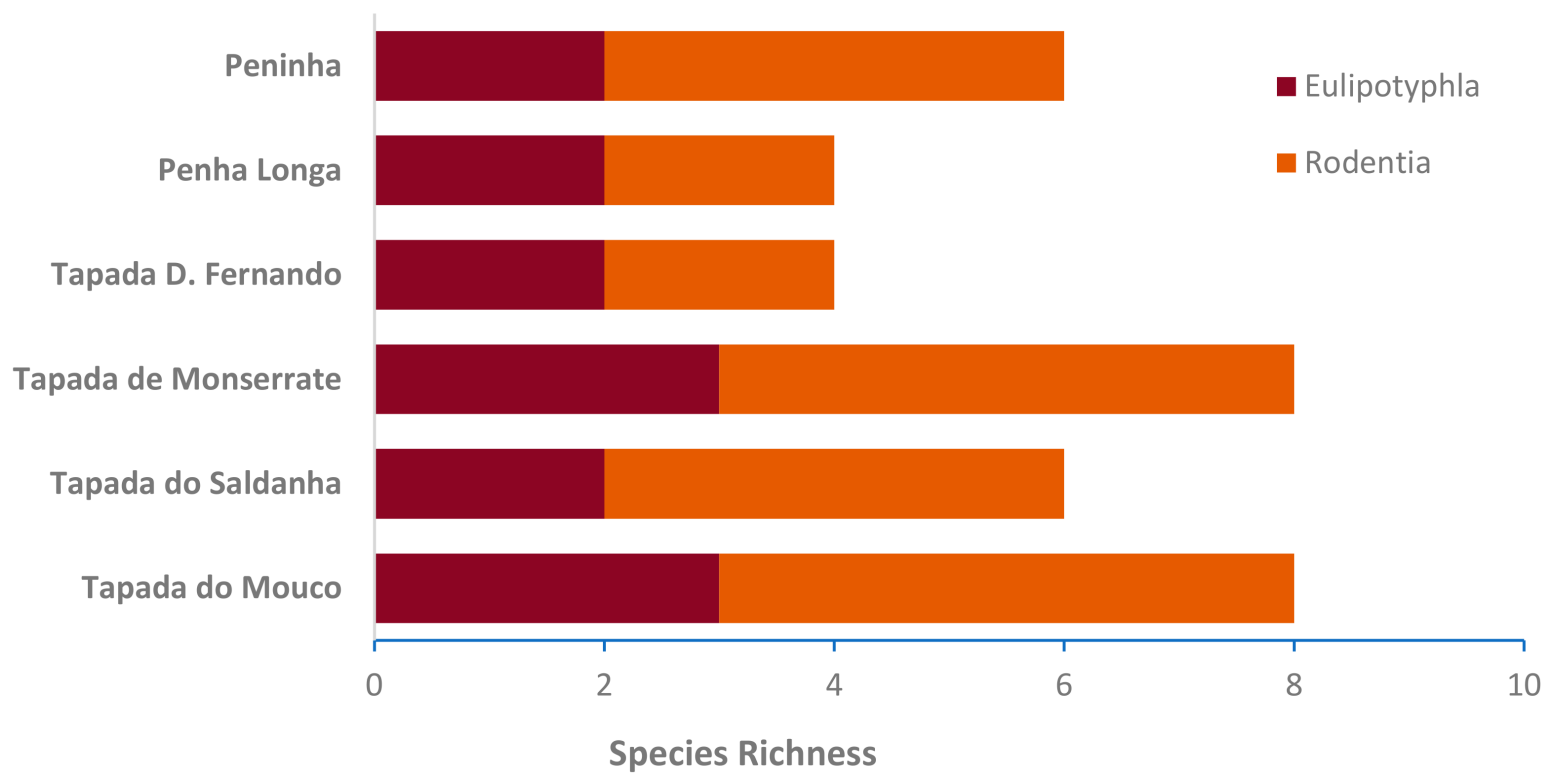
Species richness across the six sampled forest stands in Sintra-Cascais Natural Park.

In addition, at the local scale, rodents consistently exhibited higher species richness than eulipotyphlans, except for Tapada D. Fernando and Penha Longa, where rodent and shrew species richness was equivalent (Figure 5, Table 4).

Overall results showed that some of the recorded species were widely distributed across all forest stands, whereas others were more localized. The most frequently recorded species were *E. europaeus*, *C. russula*, *A. sylvaticus*, and *M. spretus*. Among these, *A. sylvaticus*, *E. europaeus*, and *C. russula* were the most widely distributed across the sampled areas. However, although well represented in the sampling, the occurrence of *M. spretus* across sites is likely underestimated due to identification uncertainty with *A. sylvaticus* in some video recordings. In contrast, a more limited number of records, and more localized occurrences, were obtained for *S. granarius*, *E. quercinus* and *M. lusitanicus* (Table 4). *T. occidentalis* and *S. vulgaris* were only occasionally recorded in both sampling periods when considering the combined trapping success of the three surveyed methods. In addition, both *Rattus norvegicus* and *Rattus rattus* are suspected to occur within the studied forested area; however, our dataset did not allow us to distinguish between the two species or confirm their respective distributions. Therefore, the data obtained were recorded as *Rattus* sp. (Table 4).

**Figure 6:**
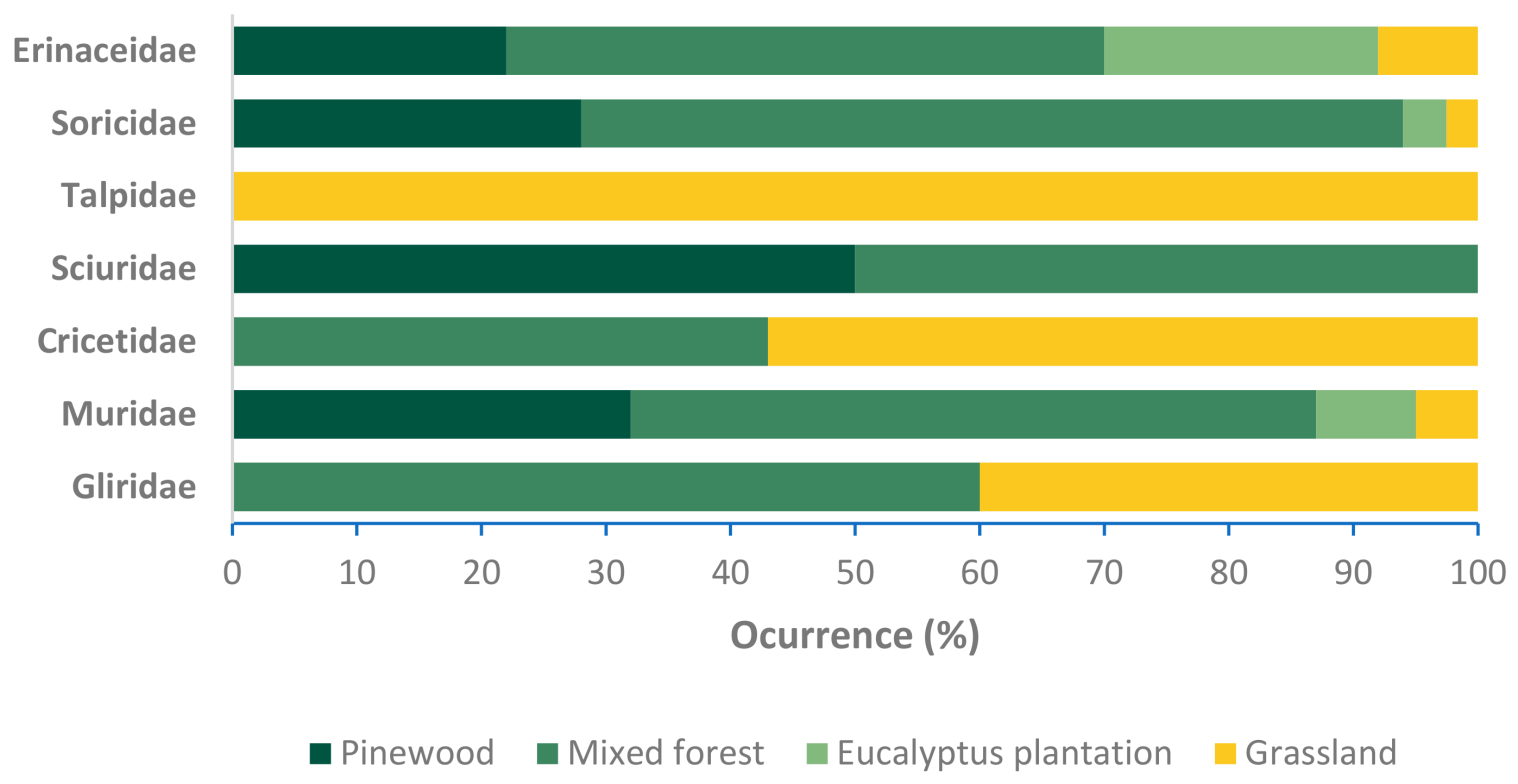
Percentage of occurrence of the different families by main habitat type across the studied area. Family members: Erinaceidae: *Erinaceus europaeus*; Soricidae: *Crocidura russula*, *Sorex granarius*; Talpidae: *Talpa occidentalis*; Gliridae: *Eliomys quercinus*; Muridae: *Apodemus sylvaticus*, *Mus spretus*, *Rattus* sp.; Cricetidae: *Microtus lusitanicus*; Sciuridae: *Sciurus vulgaris*.

Regarding species presence across surveyed habitats, it is noteworthy that the Iberian mole (*T. occidentalis*) was recorded exclusively in grasslands, whereas both the Lusitanian pine vole (*M. lusitanicus*) and the garden dormouse (*E. quercinus*) occurred in grassland and mixed forest habitats (Figure 6). The red squirrel was restricted to forested habitats. The remaining species were recorded across the range of surveyed habitat types. Of all habitat types, Eucalyptus plantations had the lowest occurrences (Figure 6).

### 3.2. Small Mammal Assessment vs. Survey Methods

Trap time-effort (in trap-nights) ranged from 15 (*C. russula*) to 480 (*S. granarius*) nights for live trapping, from 13 (*E. europaeus*) to 720 (*S. vulgaris*) nights for hair sampling, and from 5 (*A. sylvaticus*) to 1740 (*S. vulgaris*) nights for camera trapping (Table 4).

Overall, live trapping yielded the lowest inventory completeness rate for the total small mammal assemblage, whereas hair sampling detected the greatest number of species, as detailed below (Table 5).

Live trapping resulted in the inventory of six species (Table 5), three Eulipotyphla (families Soricidae, Talpidae) and three Rodentia (families Muridae, Cricetidae), represented by 79 specimens, all adults or subadults. This corresponds to a species detection rate (or trapping success per night-trap) of 3.87%, an average time-effort required for species detection of 25.8 trap-nights, and an inventory completeness rate of 60%.

Hair sampling resulted in the inventory of nine species (Table 4), three Eulipotyphla (families Erinaceidae, Soricidae) and six Rodentia (families Gliridae, Muridae, Cricetidae, Sciuridae), represented by 144 individuals. Hair sampling thus included all species detected by live trapping. This corresponds to a species detection rate of 2.91%, an average time-effort for species detection of 35 trap-nights, and an inventory completeness rate of 90%. Results suggest that hair sampling provides complementary information to live trapping, particularly by contributing to the detection of species that are more elusive, less abundant, or span a wider range of body sizes, although differences in sampling effort, location, and timing should be considered when interpreting these patterns (see Supplementary Materials S2).

Camera trapping, based on a total of 41,760 h of video recordings, resulted in the inventory of seven species (Table 5), three Eulipotyphla (families Soricidae and Erinaceidae) and four Rodentia (families Muridae, Cricetidae, and Sciuridae), represented by 569 specimen detections. This corresponded to a species detection rate of 32.70%, an average time-effort for species detection of 3.06 trap-nights, and an inventory completeness rate of 70% (Supplementary Materials S3).

These results should nevertheless be interpreted with caution, as the three survey methods were not applied uniformly across all locations and sampling periods (2018 and 2024–2025). Consequently, differences in sampling method, location, and timing may have contributed to differences in species detection and inventory completeness and should be considered when comparing the results among methods.

**Figure 7:**
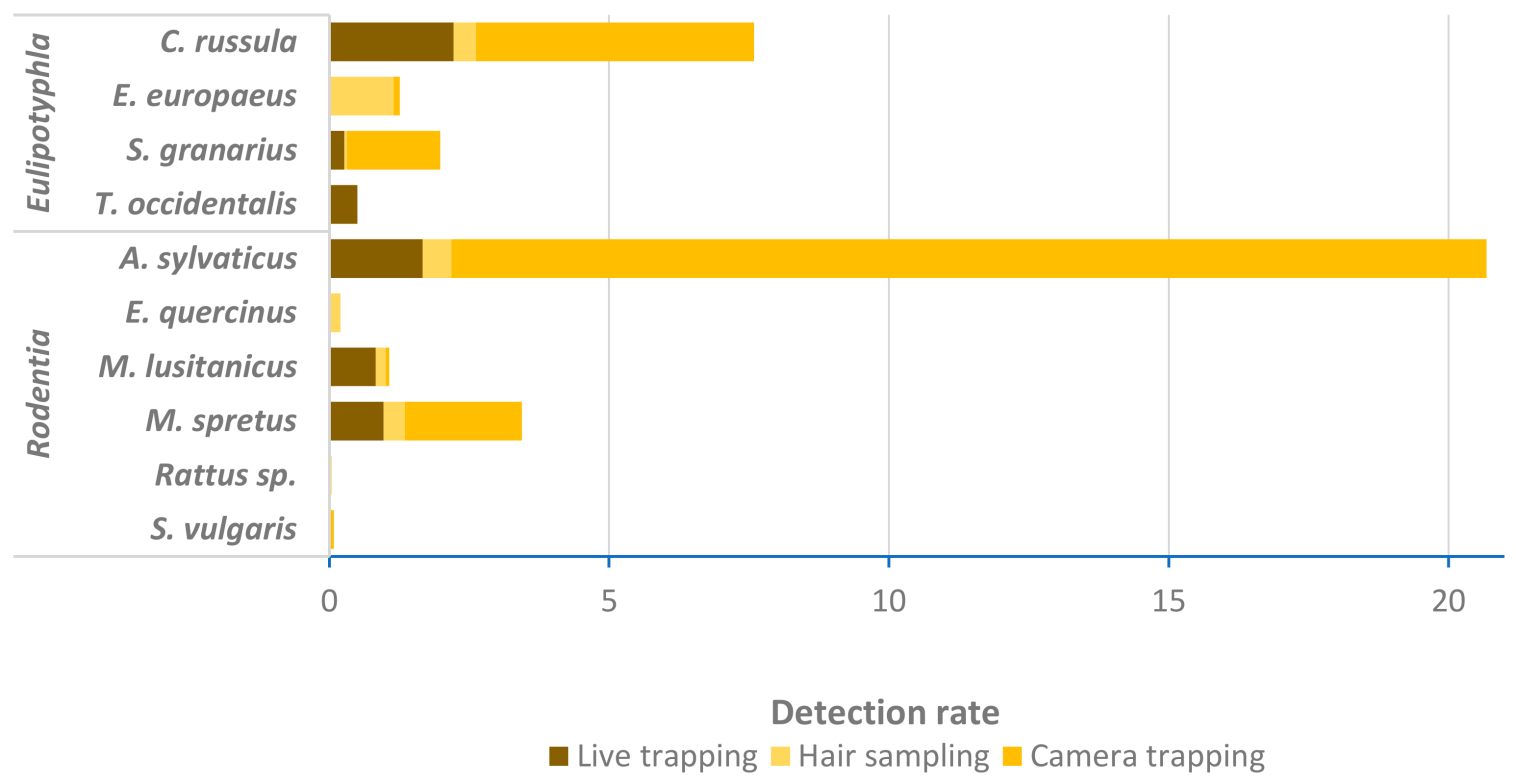
Total detection rate of the ten species inventoried in the studied forested area of the Sintra-Cascais Natural Park, using different sampling methods.

Records from the three survey methods indicate that, besides being widely distributed, the most abundant species across the study area were *C. russula*, *A. sylvaticus* and *M. spretus*—as confirmed by their relatively higher detection rates (0.4 to 4.97, 0.51 to 18.5, and 0.38 to 2.09, respectively), and the lower time-effort needed for detection (15 to 36, 5.4 to 27.7 and 34.3 to 48.3, respectively). The remaining species appear to be less abundant, including those that were only occasionally detected (*Rattus* sp., *S. vulgaris*), or require a more directed survey method (e.g., *T. occidentalis*) (Figure 7, Table 4, Supplementary Materials S3).

### 3.3. Additional Species

In addition to small mammals, other vertebrate species were detected through video recordings and/or hair samples across the surveyed locations (for location of mammals, see Table 6). These included two amphibians: the Spiny toad *Bufo spinosus* and the Common fire salamander *Salamandra salamandra* (families *Bufonidae* and *Salamandridae*, respectively); one reptile: the Iberian emerald lizard *Lacerta schreiberi* (family *Lacertidae*); three domestic and feral carnivores: the Domestic dog *Canis familiaris*, the Domestic cat *Felis catus*, and the Domestic ferret *Mustela furo* (families *Canidae*, *Felidae*, and *Mustelidae*, respectively); three wild carnivores: the Least weasel *Mustela nivalis*, the Common genet *Genetta genetta*, and the Egyptian mongoose *Herpestes ichneumon* (families *Mustelidae*, *Viverridae*, and *Herpestidae*, respectively); and one cetartiodactyl: the Wild boar *Sus scrofa* (family *Suidae*). The presence of these species should be explicitly considered in the development of any management plan focused on small mammals, particularly in light of the potential predator–prey relationships established within the park.

## 4. Discussion

The present study aimed to produce a comprehensive dataset on small mammal richness across forested areas of the Sintra-Cascais Natural Park (PNSC), within the UNESCO-designated landscape of the Sintra Mountain Range. This work represents the first systematic effort to document small mammals, and their occurrence and habitat association, across an extensive area of the PNSC, providing a robust baseline for targeted long-term ecological monitoring.

### 4.1. Species Assessment

The dataset, derived from the employment of three trapping methods—live trapping, hair sampling and camera trapping—comprises ten species, four Eulipotyphla (families Erinaceidae, Soricidae and Talpidae) and six Rodentia (families Gliridae, Muridae, Cricetidae and Sciuridae), totaling 792 records (see Supplementary Materials S1–S5).

The integration of complementary survey methods has contributed to a more robust and comprehensive assessment of small mammal communities. Each method targets distinct aspects of species detectability, and their combined use mitigates methodological bias associated with any single approach while improving overall species coverage and strengthening subsequent ecological inferences.

Live trapping enables direct capture and precise species identification. Its effectiveness, however, may be constrained by factors such as trap avoidance, saturation, bait selection, species size, and specific behavior, potentially leading to the underrepresentation of certain taxa [11,12,13]. In particular, the minced meat used as bait may have been less attractive to herbivorous species such as *Microtus lusitanicus*, for which the use of vegetable-based baits, such as carrot or natural grass, could potentially increase detection probability. Consequently, the observed occurrence and species richness may be influenced by species-specific differences in bait attractiveness. However, as alternative baits were not experimentally tested in this study, the magnitude of any such bias cannot be determined from the present data. Additionally, live trapping was generally conducted over a limited number of sampling nights, which may have further constrained species detection. These factors may have contributed to the lower number of species recorded through this method in the present study. Hair sampling provided complementary information by contributing to the detection and confirmation of species that may be difficult to capture through live trapping, including trap-averse or low-density species, while minimizing animal disturbance [14,15,16]. This is reflected in the higher number of species detected, including *Eliomys quercinus* and *Rattus* species (*Rattus norvegicus* or *Rattus rattus*), which were exclusively recorded through this method. Camera trapping provided a further complementary approach by enabling continuous, non-invasive monitoring over extended periods [17,18]. In the present survey, this approach accounted for approximately 70% of all records, although this value should not be interpreted as evidence of greater sampling performance, given that records generated by the different methods reflect different sampling designs and detection processes. Moreover, the inability to distinguish individuals means these data likely include captures and recaptures of the same animals. Additionally, depending on the quality of the video footage, some species were difficult to distinguish, resulting in some level of uncertainty compared to live trapping. Overall, these methods should therefore be regarded as complementary approaches, each providing different types of information and having distinct strengths and limitations, rather than as directly comparable measures of survey performance.

### 4.2. Species Occurrence

Patterns of species occurrence highlight the ecological success of generalist taxa, such as *Crocidura russula*, *Apodemus sylvaticus*, and *Mus spretus*, which were consistently detected across all methods and required relatively low time-effort for their detection (Figure 7; Table 4 and Table 5; Supplementary Materials S3 and S5). Their dominance and widespread occurrence likely reflect behavioral plasticity, efficient resource use, and tolerance to environmental variability [33,34,35]. In addition, increased detectability may as well be linked to frequent ground activity and opportunistic foraging, increasing encounter probabilities in trapping surveys.

In contrast, the low detection rates of *Sorex granarius*, *E. quercinus*, and *Microtus lusitanicus* underscores the combined influence of species-specific microhabitat requirements and/or methodological constraints. The restricted occurrence of *S. granarius* in localized forest patches likely reflects its dependence on stable microclimatic conditions with high humidity, which is critical for thermoregulation and water balance in shrews of the genus *Sorex* [31,36]. Similarly, the low occurrence of *E. quercinus* might reflect its general decline across the country and throughout its European range, as well as its specific habitat requirements [32]. The detection of *M. lusitanicus* in moist, herbaceous habitats aligns with its fossorial lifestyle and reliance on dense vegetation for both foraging and protection [37].

Functional traits also emerge as key determinants of detection probability. Fossorial (*Talpa occidentalis*, *M. lusitanicus*) and arboreal (*Sciurus vulgaris*) species are inherently underrepresented in conventional ground-based trapping schemes, illustrating the importance of aligning sampling design with species-specific ecological characteristics [37,38,39,40]. Likewise, larger-bodied and behaviorally cautious taxa, such as *Rattus* spp., may actively avoid traps or preferentially occupy anthropogenic environments not sampled in this study [41,42].

### 4.3. Species Habitat Association

Habitat associations further reinforce the role of ecological filtering in structuring small mammal communities (Figure 6; Supplementary Material S5). The widespread occurrence of *C. russula*, *A. sylvaticus* and *M. spretus* across forested and shrubland habitats, although lower in locations dominated by non-native species such as eucalyptus or acacia, exemplify the advantages of tolerance to habitat disturbance and the ability to exploit a wide range of trophic resources [35,43]. These species likely play significant roles in ecosystem functioning, particularly through invertebrate predation and seed dispersal. *E. europaeus* was also recorded in multiple habitat types, consistent with its generalist foraging behavior [44]. In contrast, habitat-restricted species such as *S. vulgaris* and *T. occidentalis* demonstrate strong associations with specific structural or edaphic conditions, namely tree cover and soil properties, respectively, highlighting the importance of habitat heterogeneity in maintaining functional diversity [39,40].

These findings support the notion that small mammal communities’ composition is shaped by the interplay of environmental filtering, species-specific ecological traits, and anthropogenic influences. Sintra-Cascais Natural Park offers diverse forest habitats, and it also harbors several locations dominated by eucalyptus and acacia, in which we found the most limited occurrences of small mammals across all taxa. Generalist species tend to dominate in heterogeneous or disturbed landscapes, while specialists persist in spatially limited refugia where suitable microhabitat conditions are maintained. This dynamic has important conservation implications, as ongoing landscape modification may disproportionately favor generalist taxa, leading to biotic homogenization and the erosion of specialist species [33]. Consequently, monitoring programs should integrate multi-method sampling approaches and explicitly account for detectability biases to ensure accurate assessments of community structure and biodiversity patterns.

Furthermore, the coexistence of multiple predator species across the forested areas of the park, such as the least weasel and the common genet, suggests the potential for complex intraguild interactions, including competition and predator dynamics, which can significantly modulate small mammal populations. These predators may exert both direct top-down control through predation, and indirect effects by altering behavioral and spatial responses of small mammal prey [45,46].

Overall, the information provided here constitutes a valuable basis for identifying the key environmental drivers shaping small mammal occurrence within the park and support the development and implementation of effective, integrated conservation strategies aimed at sustaining stable and functionally diverse populations over the long term.

### 4.4. Future Opportunities

Several of the sampling sites included in the present study are increasingly affected by winter storms, which produce significant alterations in the landscape, as observed during the winter of 2024. Nonetheless, such disturbances may also offer an opportunity for the organized and careful reforestation of the impacted areas, prioritizing native species in polyculture systems, such as Mediterranean forest assemblages, to promote richer, more resilient habitats with higher biodiversity.

We also recommend targeted management measures to control feral and domestic animals, particularly cats, as they can substantially increase predation pressure on small mammal populations and other wildlife. These measures should be implemented throughout the entire study area and reinforced through community awareness initiatives aimed at discouraging the free roaming of domestic pets. In areas with high human activity, especially near picnic sites and trailheads, we additionally recommend installing signage reminding visitors to keep dogs on a leash.

## 5. Conclusions

This study provides the first comprehensive assessment of small mammal communities across the principal forest habitats of the Sintra-Cascais Natural Park using a complementary combination of live trapping, hair sampling, and camera trapping. The sampling effort conducted over two survey periods generated a robust baseline dataset on species composition, distribution, relative abundance, and habitat use. The integration of different survey techniques proved effective in increasing species detectability and provided complementary information on the occurrence of small mammals within the park.

The dataset presented here contributes valuable ecological information for one of the most important protected areas in Portugal and provides an evidence-based foundation for long-term biodiversity monitoring. These results will facilitate the assessment of future environmental changes and contribute to adaptive management strategies aimed at maintaining the diversity and ecological integrity of the park’s small mammal communities. More broadly, the study demonstrates the importance of systematic, long-term monitoring for informing conservation planning and management in protected areas facing increasing anthropogenic pressures.

## Figures and Tables

**Table 1:** Description of sampled forest stands, including size, sites, forest community, and collection methods applied. Stands A–D correspond to sampling campaigns conducted in 2018, whereas stands E–F correspond to the 2024–2025 campaign.

Forest Stands (Size)	Site	Coordinates [WGS84(EPSG:4326)] (Centroid: Lat, Long)	Main Forest Community	Collection Methods		
				Live Trapping	Hair Sampling	Camera Trapping
A-Tapada do Mouco (21.3 ha)	1	38.78022, −9.40344	*Pinus pinaster*, *Cupressus lusitanica* and shrubland	x	x	
	2	38.78211, −9.40419	*Pinus pinaster*, *Cupressus lusitanica*		x	
	3	38.78211, −9.40378	Grassland	x	x	
	4	38.77836, −9.40994	*Eucalyptus globulus*, *Pinus pinaster*		x	
	5	38.78094, −9.40217	*Acacia longifolia*		x	
	6	38.78067, −9.40392	*Pinus pinaster* and grassland	x	x	
	7	38.78208, −9.40331	Grassland	x		
	8	38.78028, −9.40533	*Pinus pinaster*	x		
B-Tapada do Saldanha (20.0 ha)	9	38.76994, −9.40833	*Eucalyptus globulus*		x	
	10	38.76989, −9.40522	*Eucalyptus globulus*		x	
	11	38.76706, −9.40678	Shrubland		x	
	12	38.77433, −9.41036	*Eucalyptus globulus*	x	x	
	13	38.77556, −9.41178	*Pinus pinaster*, *Acacia longifolia*		x	
	14	38.76628, −9.40772	*Pinus pinaster*, *Eucalyptus globulus*		x	
	15	38.77672, −9.41028	*Eucalyptus globulus*	x		
	16	38.77689, −9.41172	*Pinus pinaster*	x		
	17	38.77414, −9.40831	*Eucalyptus globulus*, *Pinus pinaster*	x		
C-Tapada de Monserrate (12.8 ha)	18	38.78017, −9.42086	*Pinus pinaster*, *Quercus suber*		x	
	19	38.78336, −9.41756	*Pinus pinaster*, *Arbutus unedo*		x	
	20	38.78503, −9.41356	*Arbutus unedo*		x	
	21	38.78994, −9.42022	*Pinus pinaster* and shrubland		x	
	22	38.78783, −9.42047	*Pinus pinaster*	x	x	
	23	38.78992, −9.42139	*Arbutus unedo*	x	x	
	24	38.78900, −9.42008	*Pinus pinaster*	x		
	25	38.78864, −9.42172	*Pinus pinaster*, *Arbutus unedo*	x		
D-Tapada D.Fernando (3.4 ha)	26	38.78275, −9.43233	*Cupressus lusitanica*, *C. sempervirens*, *Pinus pinea*	x	x	
	27	38.78233, −9.43350	*Corylus avellana* and grassland	x	x	
	28	38.78403, −9.43453	*Pinus pinaster*	x	x	
	29	38.78214, −9.43692	*Pinus pinaster*	x	x	
	30	38.78267, −9.44058	*Acacia melanoxylon*, *Pinus pinaster*		x	
	31	38.78194, −9.44194	*Acacia melanoxylon*		x	
E-Penha Longa (36 ha)	32	38.76878, −9.39657	*Pinus pinaster*, *Acacia* sp.			x
	33	38.76159, −9.40742	*Eucalyptus globulus*, *Arbutus unedo*			x
F-Peninha (710 ha)	34	38.77435, −9.46615	*Acacia melanoxylon*, *Pinus pinaster*			x
	35	38.76854, −9.46956	*Quercus suber* and shrubland			x
	36	38.76874, −9.47095	*Pinus pinaster*, *Acacia longifolia*			x
	37	38.7779, −9.47243	*Cupressus lusitanica*, *Acacia melanoxylon*			x
	38	38.77074, −9.43589	*Acacia melanoxylon*			x
	39	38.77256, −9.43846	*Pinus pinea*			x
	40	38.7786, −9.46882	*Acacia melanoxylon*, *A. longifolia*, *Quercus suber*			x
	41	38.77185, −9.46666	*Fagus sylvatica*, *Abies* spp., *Quercus suber*			x
	42	38.7848, −9.46473	*Pinus pinaster*			x
	43	38.7801, −9.46871	*Eucalyptus globulus*, *Pinus pinaster*			x

**Table 2:** Small mammal species inventory of forested areas of the Sintra-Cascais Natural Park, including taxonomic identification, common names, conservation status, and number of specimens (N). Conservation categories follow the IUCN-based assessment in the *Red Book of Mammals of Portugal* (RB) [23]: LC = Least Concern; NT = Near Threatened; VU = Vulnerable; NA = Not Applicable.

ORDER/Family	Scientific Name	Common Name	RB	*N*
Eulipotyphla				
*Erinaceidae*	*Erinaceus europaeus* Linnaeus, 1758	West European Hedgehog	LC	60
Soricidae	*Crocidura russula* (Hermann 1780)	Greater White-toothed shrew	LC	135
	*Sorex granarius* Miller, 1910	Iberian shrew	VU	32
Talpidae	*Talpa occidentalis* Cabrera, 1907	Iberian mole	LC	3
Rodentia				
Gliridae	*Eliomys quercinus* (Linnaeus 1766)	European Garden dormouse	NT	10
Muridae	*Apodemus sylvaticus* (Linnaeus 1758)	Wood mouse	LC	372
	*Mus spretus* Lataste, 1883	Algerian mouse	LC	69
	*Rattus* sp.	Brown rat/Black rat	NA	2
Cricetidae	*Microtus lusitanicus* (Gerbe 1879)	Lusitanian pine vole	LC	15
Sciuridae	*Sciurus vulgaris* Linnaeus, 1758	Eurasian Red squirrel	LC	2

**Table 3:** Indices of diversity across the six sampled forest stands, with species richness (*S*), Shannon index (*H′*) and Pielou’s evenness (*J′*).

Location	*S*	*H′*	*J′*
A—Tapada do Mouco	9	1.90	0.86
B—Tapada do Saldanha	6	1.28	0.71
C—Tapada de Monserrate	8	1.32	0.64
D—Tapada D. Fernando	4	1.23	0.89
E—Penha Longa	4	0.73	0.52
F—Peninha	6	1.02	0.57

**Table 4:** Number of specimens collected (*N*) in six forest stands in Sintra-Cascais Natural Park; locations A–D correspond to sampling campaigns conducted in 2018, whereas locations E–F correspond to the 2024–2025 campaign; ‘Mice‘ corresponds to those *Apodemus sylvaticus* and *Mus spretus* that could not be distinguished.

Location	Species (*N*)	Location	Species (*N*)
A—Tapada do Mouco *N* = 101	*Erinaceus europaeus* (14)	B—Tapada do Saldanha *N* = 41	*Erinaceus europaeus* (22)
	*Crocidura russula* (14)		*Crocidura russula* (9)
	*Sorex granarius* (2)		*Eliomys quercinus* (1)
	*Talpa occidentalis* (3)		*Apodemus sylvaticus* (6)
	*Eliomys quercinus* (7)		*Mus spretus* (2)
	*Apodemus sylvaticus* (26)		*Microtus lusitanicus* (1)
	*Mus spretus* (21)		
	*Rattus* sp. (1)		
	*Microtus lusitanicus* (13)		
C—Tapada de Monserrate *N* = 40	*Erinaceus europaeus* (2)	D—Tapada D. Fernando *N* = 41	*Erinaceus europaeus* (18)
	*Crocidura russula* (25)		*Crocidura russula* (4)
	*Sorex granarius* (1)		*Apodemus sylvaticus* (13)
	*Eliomys quercinus* (2)		*Mus spretus* (6)
	*Apodemus sylvaticus* (5)		
	*Mus spretus* (3)		
	*Rattus* sp. (1)		
	*Sciurus vulgaris* (1)		
E—Penha Longa *N* =130	*Erinaceus europaeus* (2)	F—Peninha *N* = 439	*Crocidura russula* (76)
	*Crocidura russula* (10)		*Sorex granarius* (29)
	*Apodemus sylvaticus* (76)		*Apodemus sylvaticus* (246)
	*Mus spretus* (9)		*Mus spretus* (27)
	Mice (33)		*Microtus lusitanicus* (1)

**Table 5:** Trap time-effort (in trap-nights) and inventory completeness rate across different trapping methods. Trap time-effort considers total trapping effort and total number of small mammals detected in the six forest stands; ‘Mice‘ corresponds to those *Apodemus sylvaticus* and *Mus spretus* that could not be distinguished; species that consistently required less time for detection are indicated in bold.

Order/Species	Live Trapping (4 Forest Stands)	Hair Sampling (4 Forest Stands)	Camera Trapping (2 Forest Stands)
Eulipotyphla			
*Erinaceus europaeus*	-	13	870
*Crocidura russula*	15	36	20
*Sorex granarius*	480	360	60
*Talpa occidentalis*	160	-	-
Rodentia			
*Eliomys quercinus*	-	72	-
*Apodemus sylvaticus*	20	28	5
*Mus spretus*	34	38	48
*Rattus* sp.	-	360	-
Mice	-	-	19
*Microtus lusitanicus*	96	80	1740
*Sciurus vulgaris*	-	720	1740
Inventory completeness rate	60%	90%	70%

**Table 6:** Additional wild mammals recorded across forested areas of Sintra-Cascais Natural Park.

Species Scientific Name	Common Name	Forest Stand and SITE
*Mustela nivalis* Linnaeus, 1766	Least weasel	Tapada do Mouco: Site 2 Peninha: Site 36
*Genetta genetta* (Linnaeus, 1758)	Common genet	Penha Longa: Sites 32, 33 Peninha: Sites 37, 38, 39, 40, 43
*Herpestes ichneumon* (Linnaeus, 1758)	Egyptian mongoose	Penha Longa: Site 32
*Sus scrofa* Linnaeus, 1758	Wild boar	Peninha: Site 40

## Data Availability

The original contributions of this study are included in the article and its Supplementary Materials. Approximately 40,000 h of video recordings, containing detailed information on species presence, distribution, and diel activity, are archived and available for more in-depth analysis. Additional details about the data presented in this study can be obtained from the corresponding authors upon request.

## Supplementary Materials

The following supporting information can be downloaded at: https://www.mdpi.com/article/10.3390/ani16162590/s1, S1: Location and site characterization, Figure in S1. Study area and species occurrences; S2: Trapping success vs. trapping methods, Table in S2. Trapping success in the six surveyed forested stands; S3: Species independent detection per trap-night vs. trapping methods, Figures in S3. Species detection rate; S4: Photo-records of the species inventoried through camera trapping, Figure in S4. Species photo-records; S5: Occurrence maps of the ten small mammal species inventoried in Sintra-Cascais Natural Park, Maps in S5. Species occurrence maps.
