# Supplementary material for: Assessment of Small Mammal Communities in Forested Areas of the Sintra-Cascais Natural Park (Portugal)
Source: Animals (Basel). 2026 Aug 19;16(16):2590. doi: 10.3390/ani16162590 (PMC13508988; doi:10.3390/ani16162590)
Supplement: Supplementary file 1 [file animals-16-02590-s001.zip › Supplementary Material project report.pdf]

Assessment of Small Mammal Communities in Forested Areas of the Sintra-Cascais Natural Park (Portugal)

Joaquim T. Tapisso, Rita I. Monarca, Ana M. Cerveira, Sophie von Merten, Maílis Carrilho, Guilherme Aparício, Maria da Graça Ramalhinho, Maria da Luz Mathias

Suppl. material S1: Study area, main forest communities and species occurrences; Locations A–D correspond to sampling campaigns conducted in 2018, whereas locations E–F correspond to campaigns conducted in 2024–2025. 'Mice' signifies undistinguished *Apodemus sylvaticus* and *Mus spretus*.

A-Tapada do Mouco

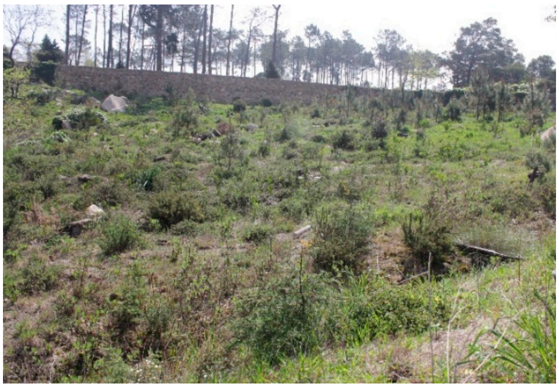

Site 1. 38.78022, -9.40344
Main forest community*:* *Pinus pinaster, Cupressus lusitanica* and shrubland
Small mammals: *Erinaceus europaeus*, *Eliomys quercinus*, *Apodemus sylvaticus*, *Mus spretus*, *Rattus* sp., *Microtus lusitanicus*

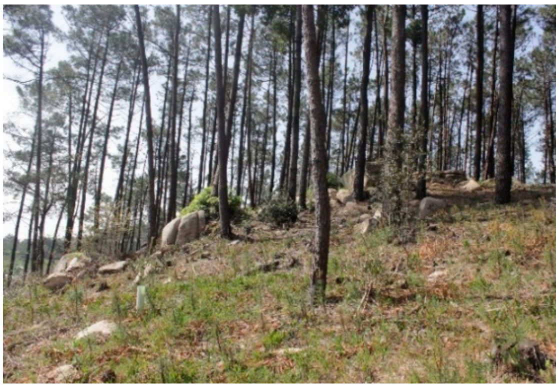

Site 2. 38.78211, -9.40419
Main forest community*:* *Pinus pinaster, Cupressus lusitanica*
Small mammals: *E.europaeus*, *M. spretus*

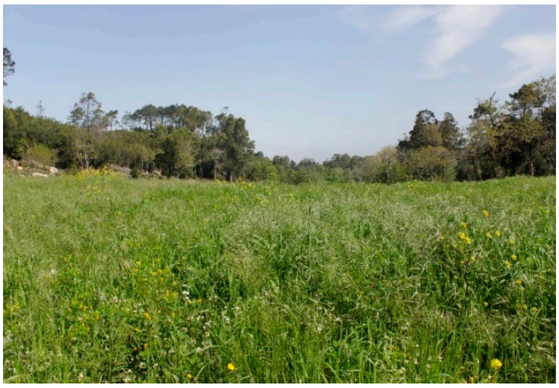

Site 3: 38.78211, -9.40378
Main forest community*:* Grassland
Small mammals: *E.europaeus*, *Crocidura russula*, *Sorex granarius*, *Talpa occidentalis, E.quercinus*, *A.sylvaticus, M.spretus*, *M.lusitanicus*

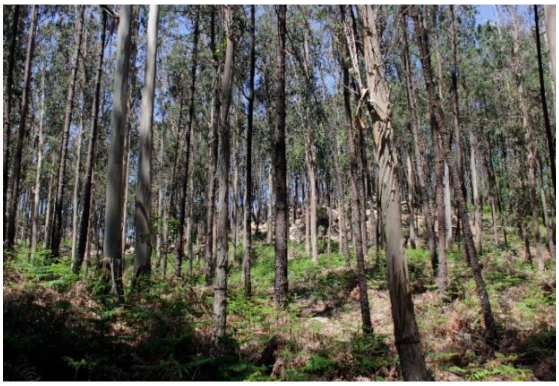

Site 4: 38.77836, -9.40994
Main forest community*:* *Eucalyptus globulus, Pinus pinaster*
Small mammals: *C.russula*, *M.spretus*

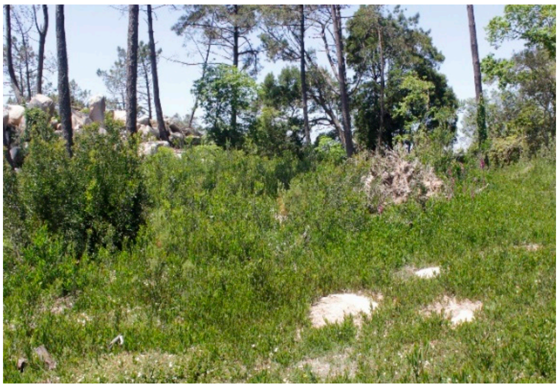

Site 5: 38.78094, -9.40217
Main forest community*:* *Acacia longifolia*
Small mammals: *E.europaeus*, *M.spretus*

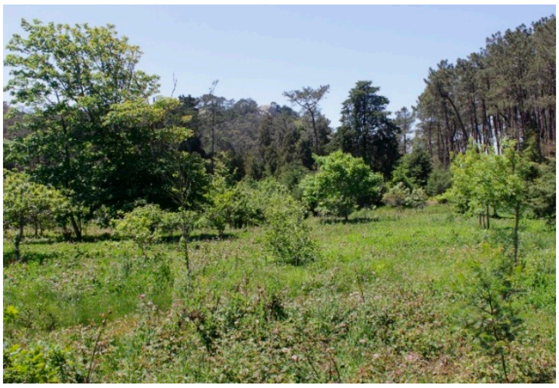

Site 6: 38.78067, -9.40392
Main forest community*:* *Pinus pinaster* and grassland
Small mammals: *E.europaeus*, *C.russula*, *S.granarius*, *A.sylvaticus*, *M.spretus*

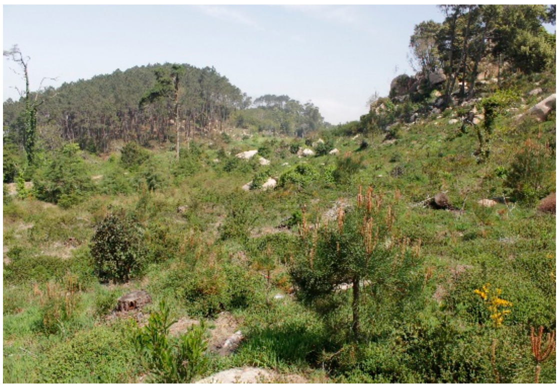

Site 7: 38.78208, -9.40331
Main forest community*:* Grassland
Small mammals: *C.russula*, *A.sylvaticus*, *M.spretus*

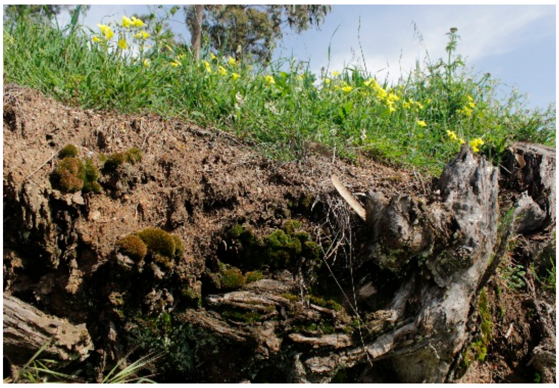

Site 8: 38.78028, -9.40533
Main forest community*:* *Pinus pinaster*
Small mammals: *C.russula*

B - Tapada do Saldanha

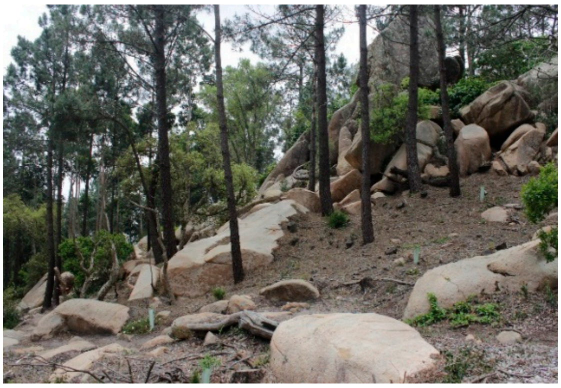

Site 9: 38.76994, -9.40833
Main forest community*:* *Eucalyptus globulus*
Small mammals: *E.europaeus*

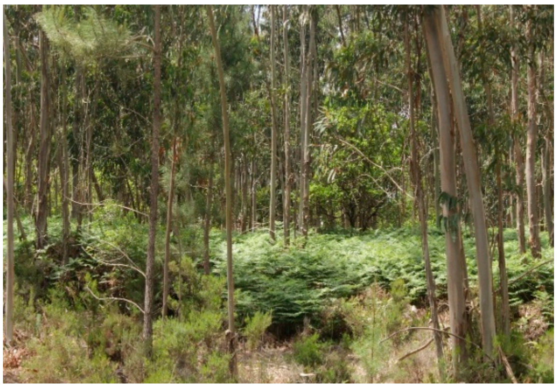

Site 10: 38.76989, -9.40522
Main forest community*:* *Eucalyptus globulus*
Small mammals: *E.europaeus*

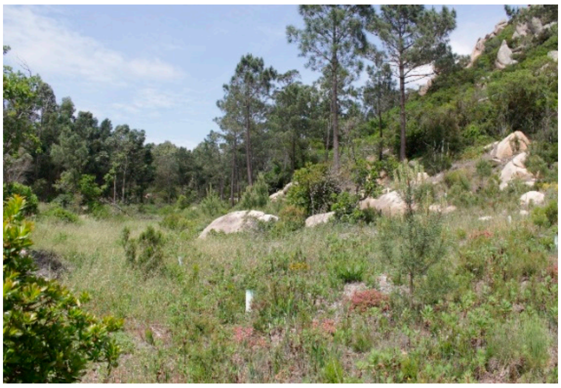

Site 11- 38.76706, -9.40678
Main forest community*:* Shrubland
Small mammals: *E.europaeus*, *M.lusitanicus*

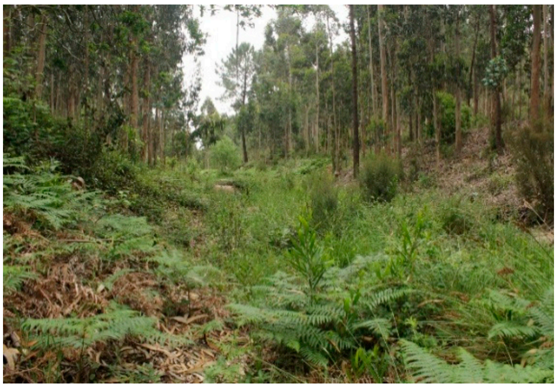

Site 12: 38.77433, -9.41036
Main forest community*:* *Eucalyptus globulus*
Small mammals: *E.europaeus*, *C.russula*, *A.sylvaticus*, *M.spretus*

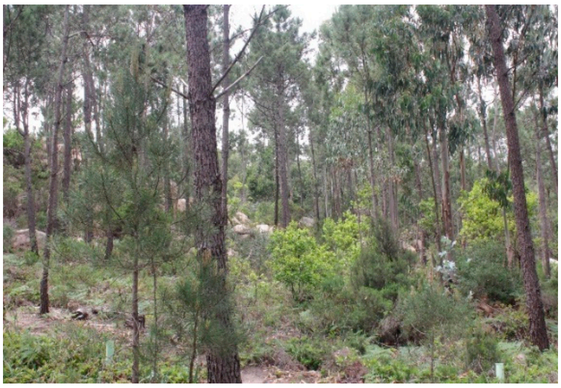

Site 13: 38.77556, -9.41178
Main forest community*:* *Pinus pinaster, Acacia longifolia*
Small mammals: *E.europaeus*

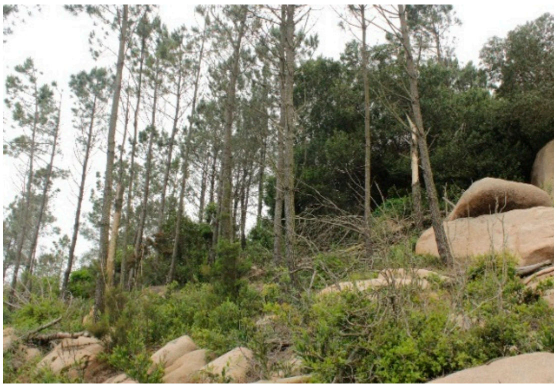

Site 14: 38.76628, -9.40772
Main forest community*:* *Pinus pinaster, Eucalyptus globulus*
Small mammals: *E.europaeus*, *E. quercinus*

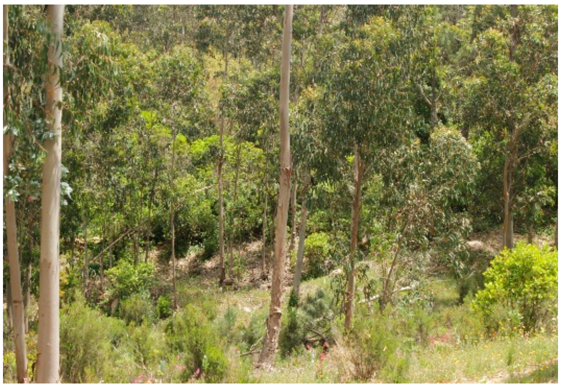

Site 15: 38.77672, -9.41028
Main forest community*:* *Eucalyptus globulus*
Small mammals: *C.russula*, *A.sylvaticus*, *M.spretus*

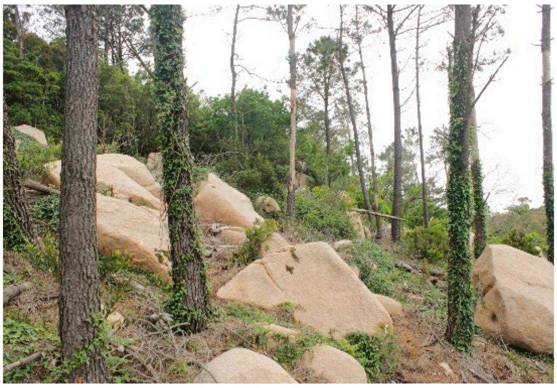

Site 16 - 38.77689, -9.41172
Main forest community*:* *Pinus pinaster*
Small mammals: *C.russula*

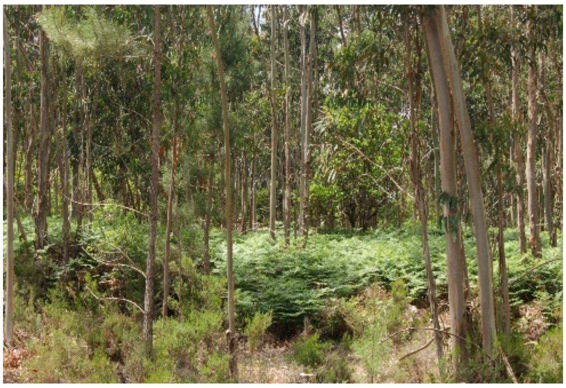

Site 17: 38.77414, -9.40831
Main forest community*:* *Eucalyptus globulus, Pinus pinaster*
Small mammals: *C.russula*

C - Tapada de Monserrate

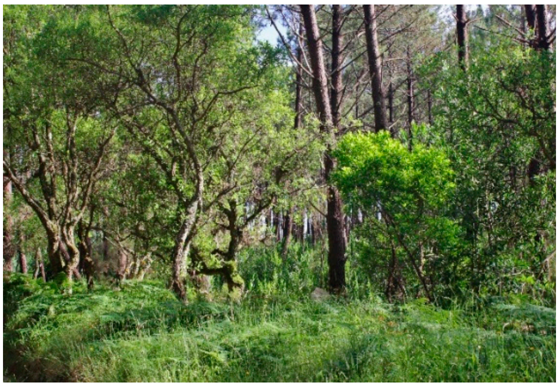

Site 18: 38.78017, -9.42086
Main forest community*:* *Pinus pinaster*, *Quercus suber*
Small mammals: *C.russula*, *A.sylvaticus*

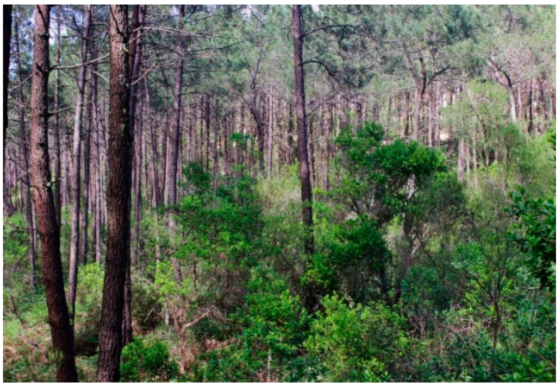

Site 19: 38.78336, -9.41756
Main forest community*:* *Pinus pinaster, Arbustus unedo*
Small mammals: *C.russula*, *S. granarius*

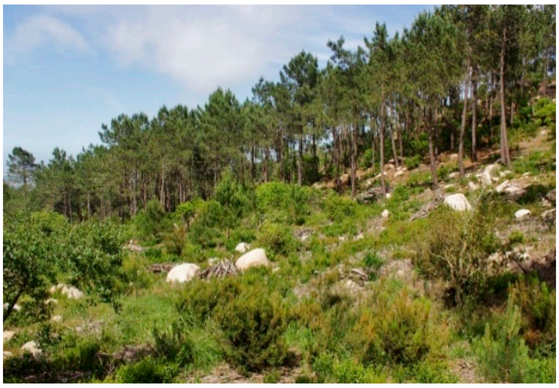

Site 20: 38.78503, -9.41356
Main forest community*:* *Arbustus unedo*
Small mammals: *E.europaeus*

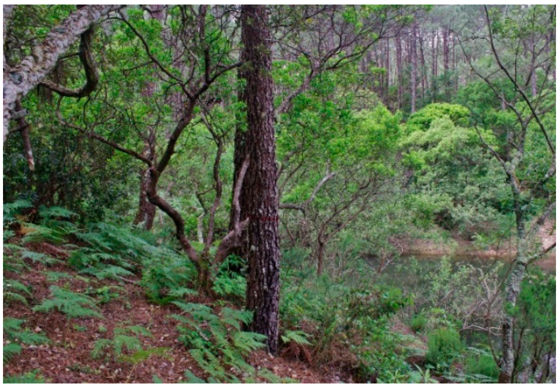

Site 21: 38.78994, -9.42022
Main forest community*:* *Pinus pinaster* and shrubland
Small mammals: *C.russula*, *A.sylvaticus*, *M.spretus*

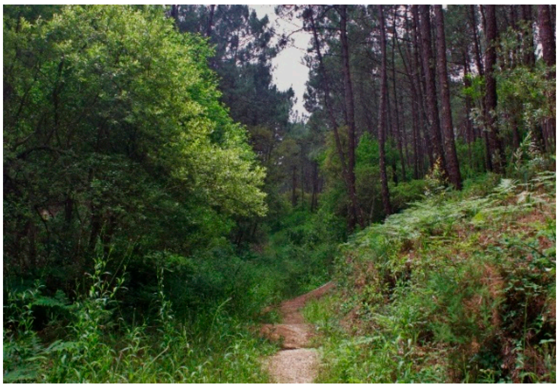

Site 22: 38.78783, -9.42047
Main forest community*:* *Pinus pinaster*
Small mammals: *E.europaeus*, *C.russula*, *A.sylvaticus*

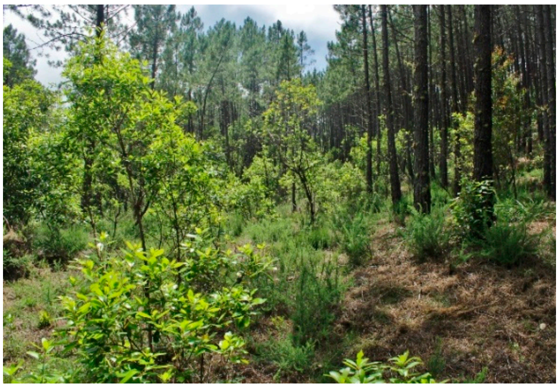

Site 23: 38.78992, -9.42139
Main forest community*:* *Arbustus unedo*
Small mammals: *E.europaeus*, *C.russula*, *A.sylvaticus*, *Rattus* sp., *Sciurus vulgaris*

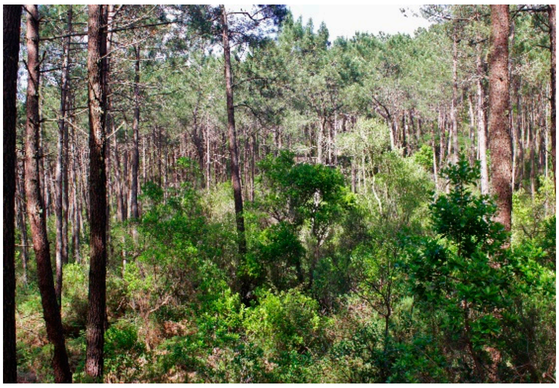

Site 24: 38.78900, -9.42008
Main forest community*:* *Pinus pinaster*
Small mammals: *E.europaeus*, *C.russula*, *A.sylvaticus*

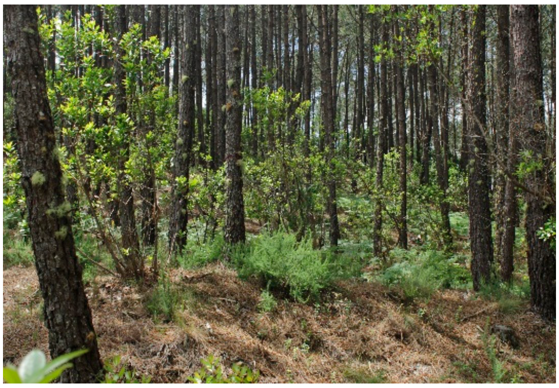

Site 25: 38.78864, -9.42172
Main forest community*:* *Pinus pinaster, Arbustus unedo*
Small mammals: *C.russula*, *E.quercinus*, *A.sylvaticus*, *Rattus* sp., *Sciurus vulgaris*

D - Tapada D. Fernando

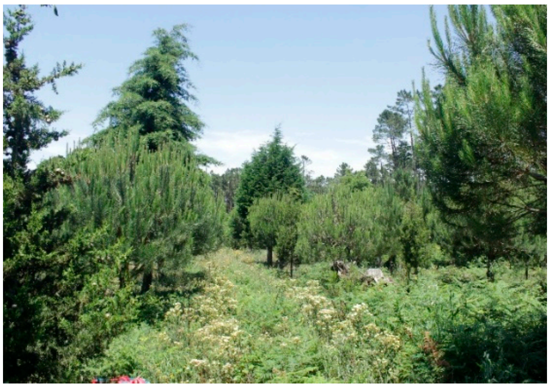

Site 26: 38.78275, -9.43233
Main forest community*:* *Cupressus lusitanica, C. sempervirens, Pinus pinea*
Small mammals: *C.russula*, *A.sylvaticus*

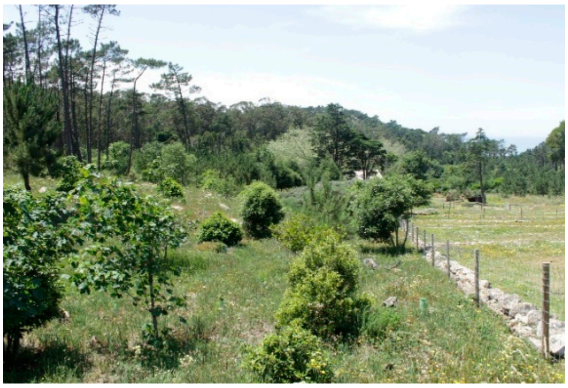

Site 27: 38.78233, -9.43350
Main forest community*:* *Corylus avellana* and grassland
Small mammals: *E.europaeus*, *C.russula*, *A.sylvaticus*, *Mus spretus*

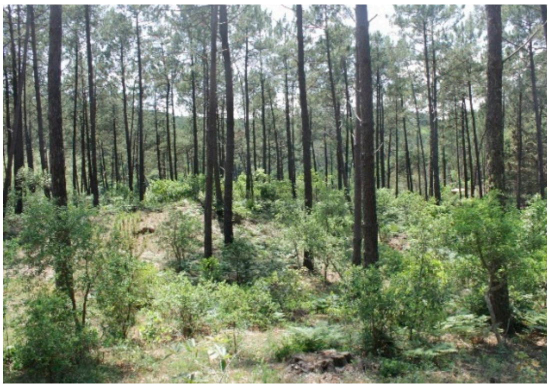

Site 28: 38.78403, -9.43453
Main forest community*:* *Pinus pinaster*
Small mammals: *E.europaeus*

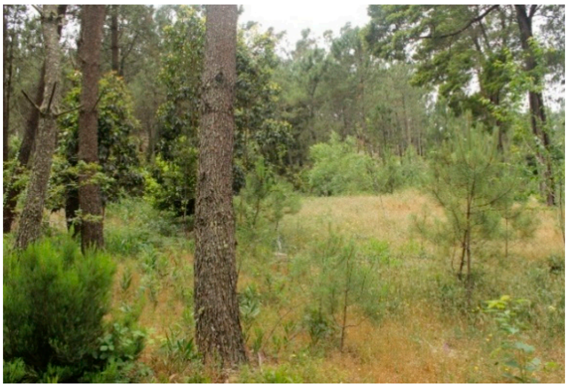

Site 29: 38.78214, -9.43692
Main forest community*:* *Pinus pinaster*
Small mammals: *E.europaeus, C.russula, A.sylvaticus*

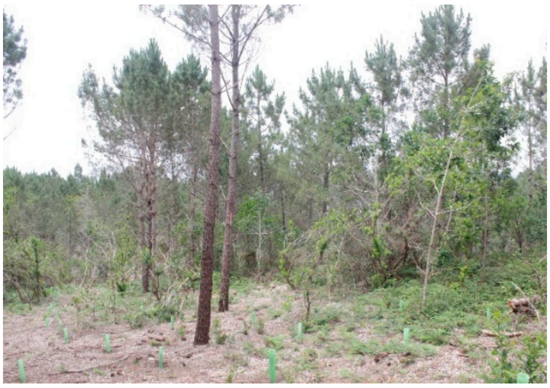

Site 30: 38.78267, -9.44058
Main forest community*:* *Acacia melanoxylon, Pinus pinaster*
Small mammals: *A.sylvaticus*

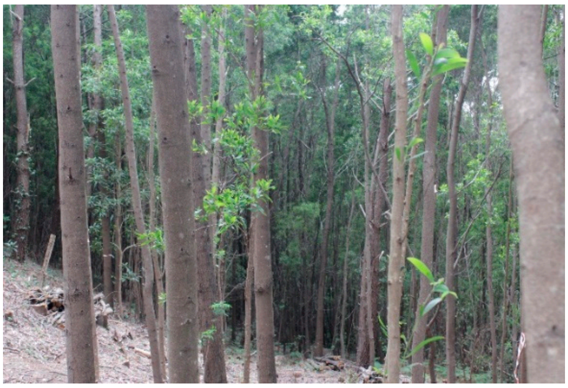

Site 31: 38.78194, -9.44194
Main forest community*:* *Acacia melanoxylon*
Small mammals: *E.europaeus, C.russula, A.sylvaticus*

E - Penha Longa

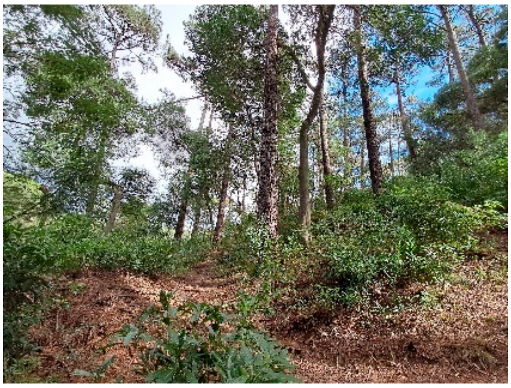

Site 32: 38.76878, -9.39657
Main forest community*:* *Pinus pinaster* and *Acacia sp.*
Small mammals: *E.europaeus, C.russula, A.sylvaticus, M.spretus,* Mice

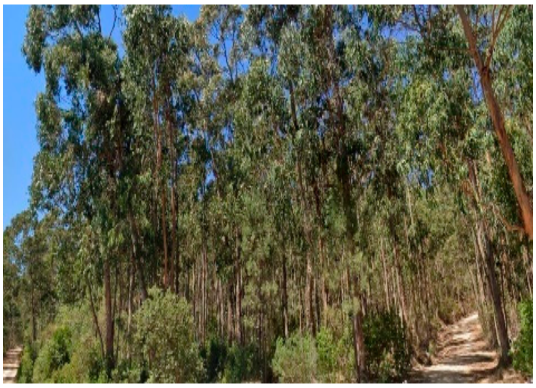

Site 33*:* 38.76159, -9.40742
Main forest community*:* *Eucalyptus globulus* and *Arbutus unedo*
Small mammals: *C.russula, A.sylvaticus,* Mice

F - Peninha

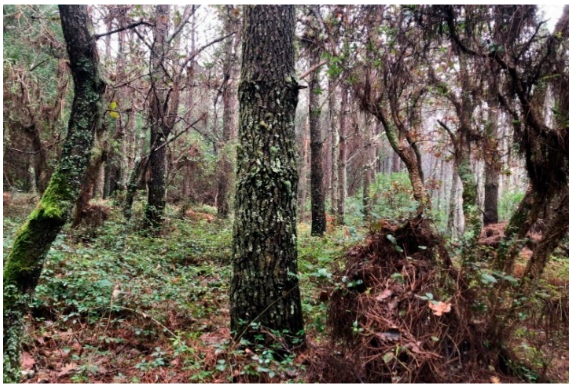

Site 34: 38.77435, -9.46615
Main forest community: *Acacia melanoxylon* and *Pinus pinaster*
Small mammals: *C.russula, A.sylvaticus,* Mice

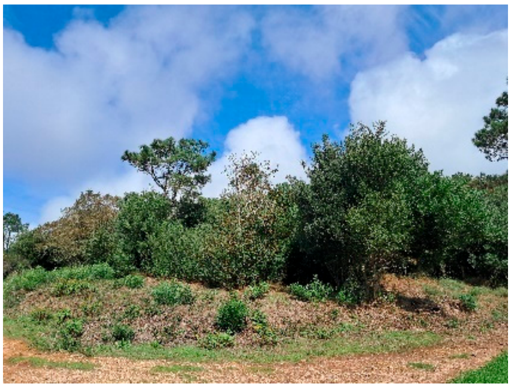

Site 35: 38.76854, -9.46956
Main forest community: *Quercus suber* and Shrubland
Small mammals: *C.russula, S. granarius, A.sylvaticus,* Mice

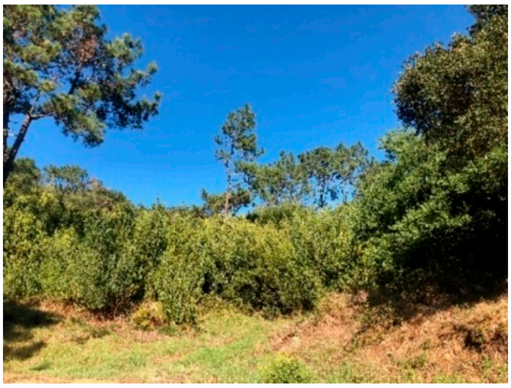

Site 36: 38.76874, -9.47095
Main forest community: *Pinus pinaster* and *Acacia longifolia*
Small mammals: *C.russula, S.granarius, A.sylvaticus, M.spretus,* Mice, *M. lusitanicus*

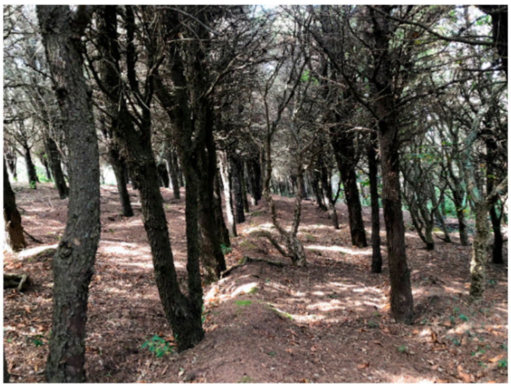

Site 37: 38.7779, -9.47243
Main forest community: *Cupressus lusitanica* and *Acacia melanoxylon*
Small mammals: *C.russula, S. granarius, A.sylvaticus, M.spretus,* Mice

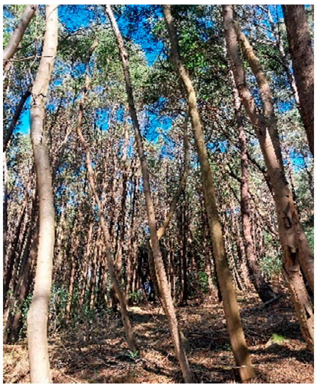

Site 38: 38.77074, -9.43589
Main forest community: *Acacia melanoxylon*
Small mammals: *C.russula, A.sylvaticus*

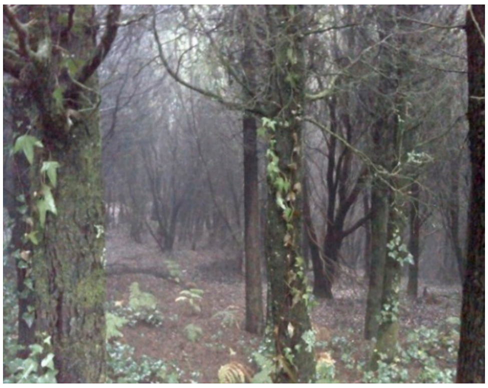

Site 39: 38.77256, -9.43846
Main forest community: *Pinus pinea*
Small mammals: *C.russula, A.sylvaticus,* Mice, *S. vulgaris*

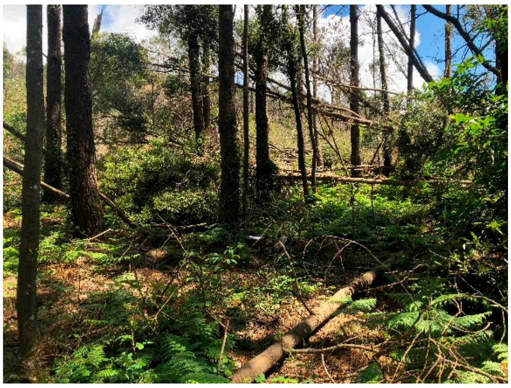

Site 40: 38.7786, -9.46882
Main forest community: *Acacia melanoxylon, Acacia longifolia* and *Quercus suber*
Small mammals: *C.russula, A.sylvaticus,* Mice

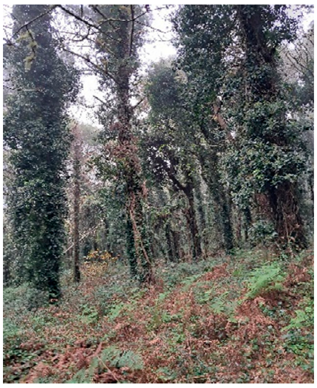

Site 41: 38.77185, -9.46666
Main forest community*:* *Fagus sylvatica, Abies sp.* and *Quercus suber*
Small mammals: *C.russula, S. granarius, A.sylvaticus,* Mice

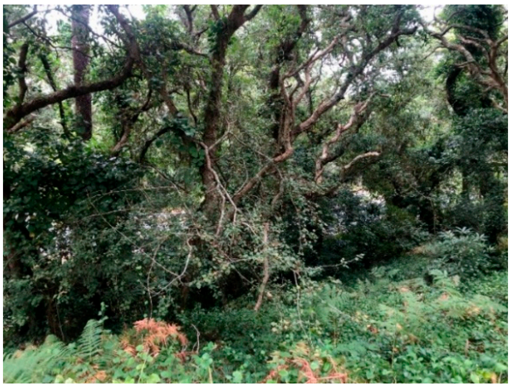

Site 42: 38.7848, -9.46473
Main forest community*:* *Pinus pinaster*
Small mammals: *C.russula, S.granarius, A.sylvaticus,* Mice

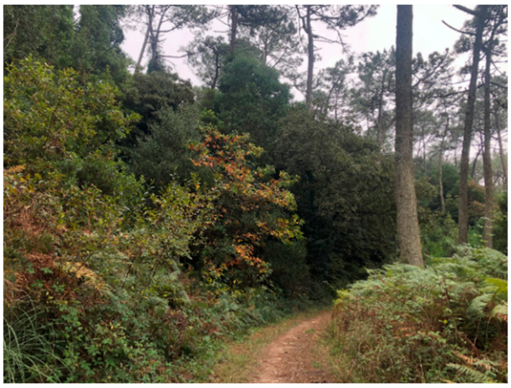

Site 43: 38.7801, -9.46871
Main forest community*:* *Eucalyptus globulus* and *Pinus pinaster*
Small mammals: *C.russula, S.granarius, A.sylvaticus,* Mice

Suppl. material S2: Trapping success vs. trapping methods across the six surveyed forested stands in the Sintra-Cascais Natural Park; number of specimens caught are indicated within brackets; N-total number of specimens in each location

Authors: Joaquim Tapisso, Rita I. Monarca, Ana M. Cerveira, Sophie von Merten, Maílis Carrilho, Guilherme Aparício, Maria da Graça Ramalhinho, Maria da Luz Mathias

Data type: Trapping success vs. trapping methods

Brief description: Species and numbers caught at each surveyed location, using different trapping methods: live-trapping, hair sampling, camera trapping.

**Location**	**Sampling method**		
	Live trapping	Hair sampling	Camera trapping
**Tapada do Mouco N=101**	*Crocidura russula* (8)<br>*Sorex granarius* (1)<br>*Talpa occidentalis* (3)<br>*Apodemus sylvaticus* (5)<br>*Mus spretus* (6)<br>*Microtus lusitanicus* (5)	*Erinaceus europaeus* (14)<br>*Crocidura russula* (6)<br>*Sorex granarius* (1)<br>*Eliomys quercinus* (7)<br>*Apodemus sylvaticus* (21)<br>*Mus spretus* (15)<br>*Rattus sp.* (1)<br>*Microtus lusitanicus* (8)	
**Tapada do Saldanha N=41**	*Crocidura.russula* (8)<br>*Apodemus sylvaticus* (5)<br>*Mus spretus* (1)	*Erinaceus europaeus* (23)<br>*Crocidura russula* (1)<br>*Apodemus sylvaticus* (1)<br>*Mus spretus* (1)<br>*Microtus lusitanicus* (1)	
**Tapada de Monserrate N=40**	*Crocidura russula* (14)<br>*Apodemus sylvaticus* (3)<br>*Mus spretus* (1)	*Erinaceus europaeus* (2)<br>*Crocidura russula* (11)<br>*Sorex granarius* (1)<br>*Eliomys quercinus* (2)<br>*Apodemus sylvaticus* (2)<br>*Mus spretus* (2)<br>*Rattus sp.* (1)<br>*Sciurus vulgaris* (1)	
**Tapada D.Fernando N=41**	*Crocidura russula* (2)<br>*Apodemus sylvaticus* (11)<br>*Mus spretus* (6)	*Erinaceus europaeus* (18)<br>*Crocidura russula* (2)<br>*Apodemus sylvaticus* (2)	
**Penha Longa N=130**			*Erinaceus europaeus* (2)<br>*Crocidura russula* (10)<br>*Apodemus sylvaticus* (76)<br>*Mus spretus* (9)<br>Mice (33)
**Peninha N=439**			*Crocidura russula* (76)<br>*Sorex granarius* (29)<br>*Apodemus sylvaticus* (246)<br>*Mus spretus* (27)<br>*Microtus lusitanicus* (1)<br>*Sciurus vulgaris* (1)<br>Mice (59)

Suppl. material S3: Independent detections per species per trap-night vs. trapping methods

Authors: Joaquim Tapisso, Rita I. Monarca, Ana M. Cerveira, Sophie von Merten, Maílis Carrilho, Guilherme Aparício, Maria da Graça Ramalhinho, Maria da Luz Mathias

Data type: Species detection rate

Brief description: Species detection rate, reflecting relative abundances, in the six inventoried locations, using different trapping methods: live-trapping, hair sampling, camera trapping.

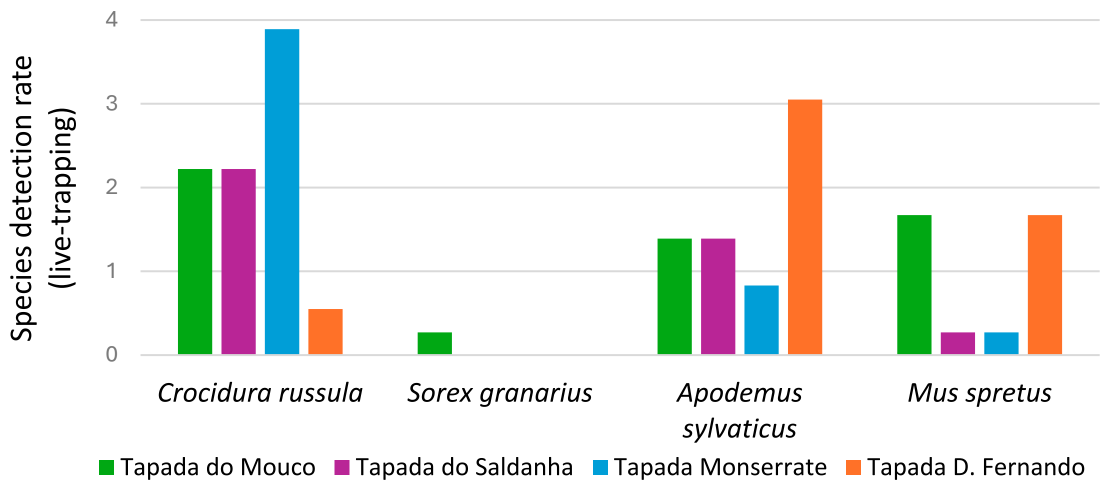

Suppl. material S3.1. Species detection rate, reflecting live-trapping success per unit of effort in four forest stands during the 2018 campaign.

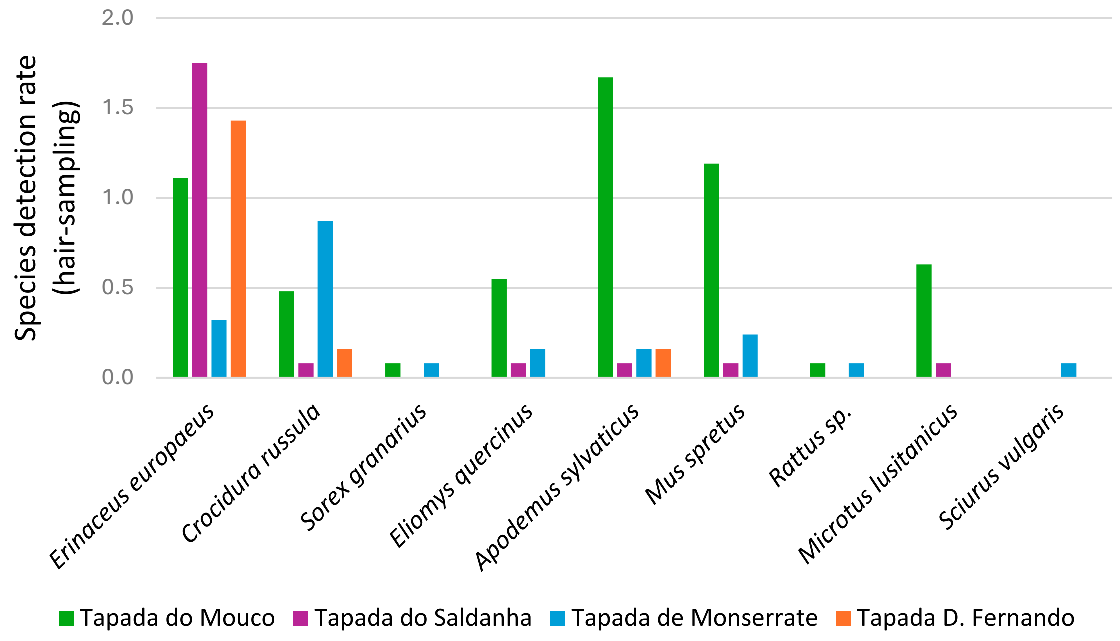

Suppl. material S3.2. Species detection rate reflecting hair-sampling success per unit of effort in four forest stands and 31 sites during the 2018 campaign.

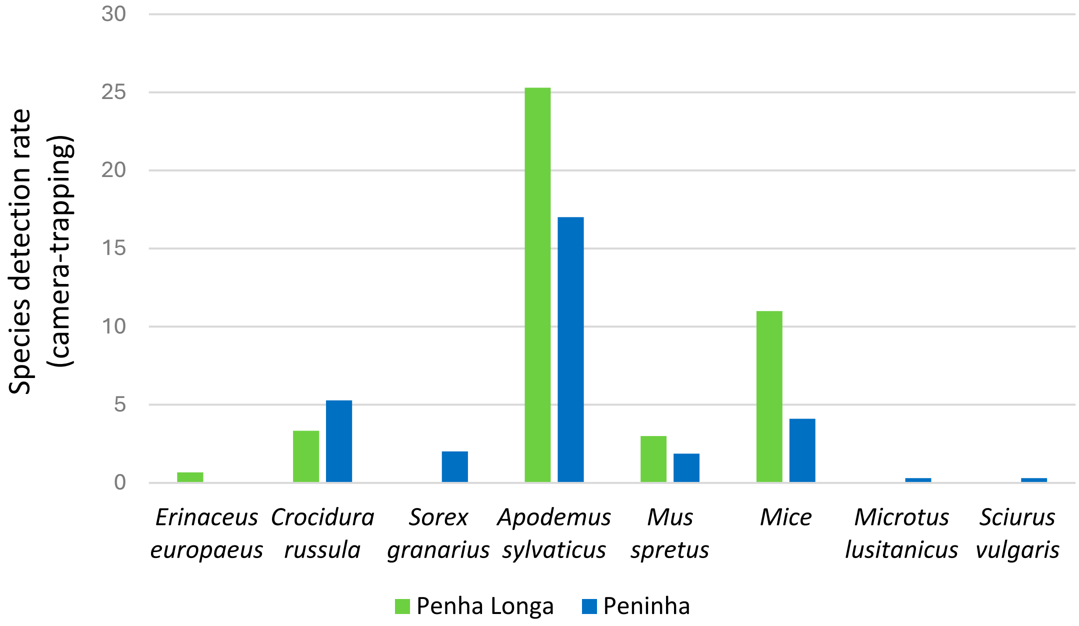

Suppl. material S3.3. Species detection rate, reflecting camara-trapping success per unit of effort, in two forest stands and 12 sites, during the 2024-2025 campaign.

Suppl. Material S4: Photo-records of the species inventoried through camera trapping.

Authors: Joaquim Tapisso, Rita I. Monarca, Ana M. Cerveira, Sophie von Merten, Maílis Carrilho, Guilherme Aparício, Maria da Graça Ramalhinho, Maria da Luz Mathias

Data type: Species photo-records

Brief description: Images of the small mammal species identified through camera trapping

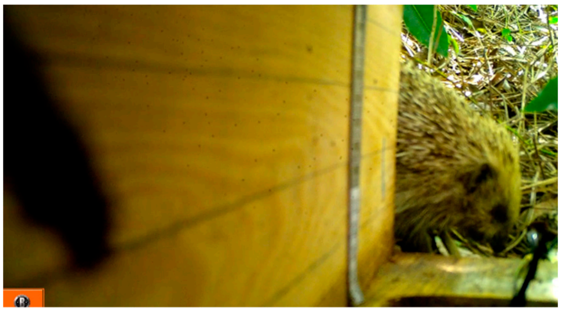

Erinaceus europaeus

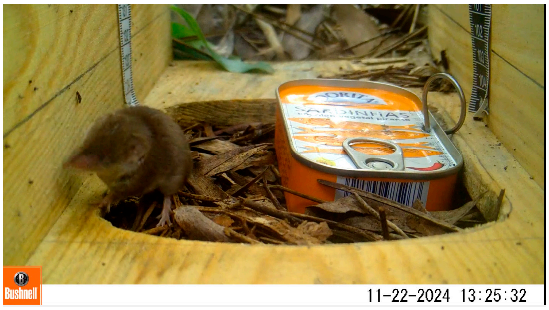

Crocidura russula

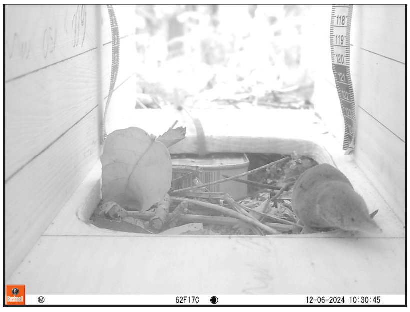

Sorex granarius

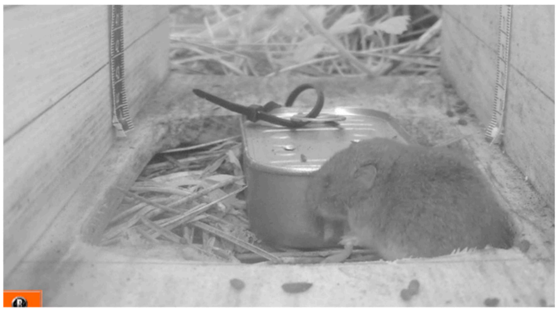

Microtus lusitanicus

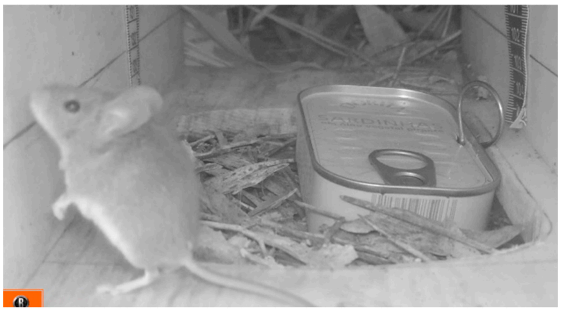

Apodemus sylvaticus

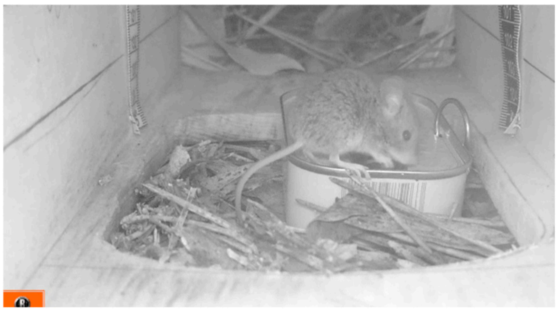

Mus spretus

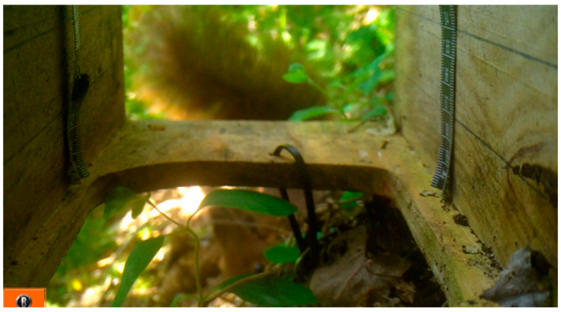

Sciurus vulgaris

Suppl. material S5: Occurrence maps of the ten small mammal species inventoried in Sintra-Cascais Natural Park

Authors: Joaquim Tapisso, Rita I. Monarca, Ana M. Cerveira, Sophie von Merten, Maílis Carrilho, Guilherme Aparício, Maria da Graça Ramalhinho, Maria da Luz Mathias

Data type: Species occurrence maps

Brief description: Occurrence maps of the four Eulipotyphla (families Erinaceidae, Soricidae, Talpidae) and six Rodentia species (families Gliridae, Muridae, Cricetidae, Sciuridae) inventoried in forest stands in the Sintra-Cascais Natural Park

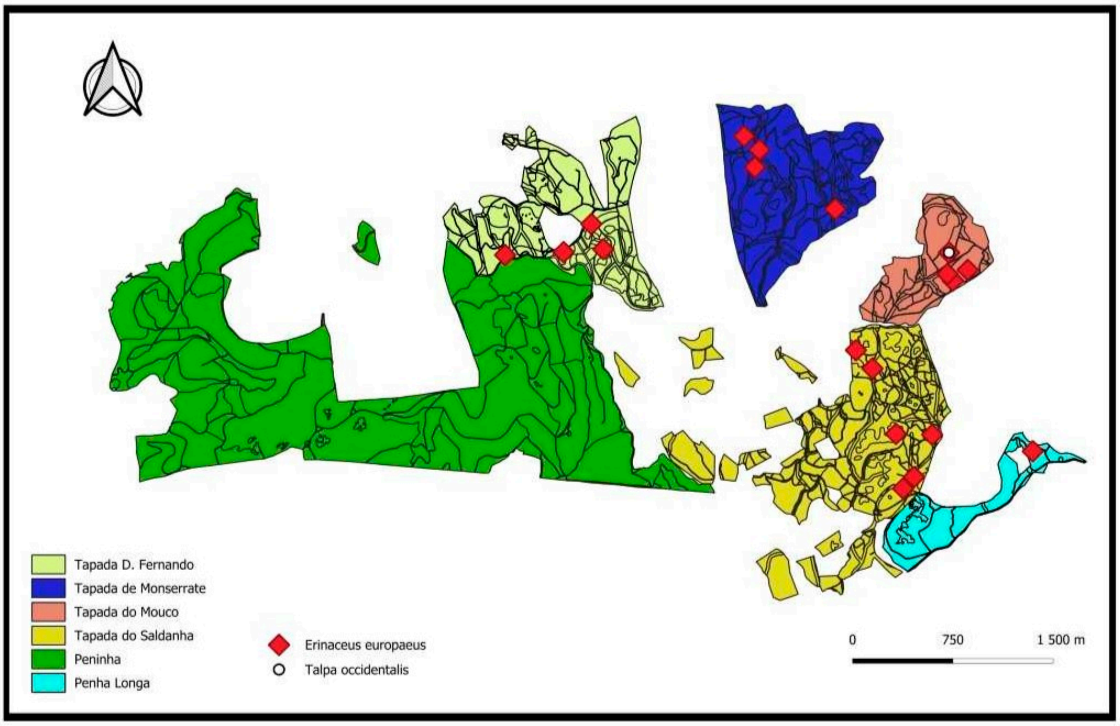

Suppl. material S5.1. Occurrence of *Erinaceus europaeus* (family Erinaceidae) and *Talpa occidentalis* (family Talpidae) over six forest stands in the Sintra-Cascais Natural Park; species presences correspond to sites surveyed in each location as indicated in Suppl. material S1 and Figure 1 in the main text.

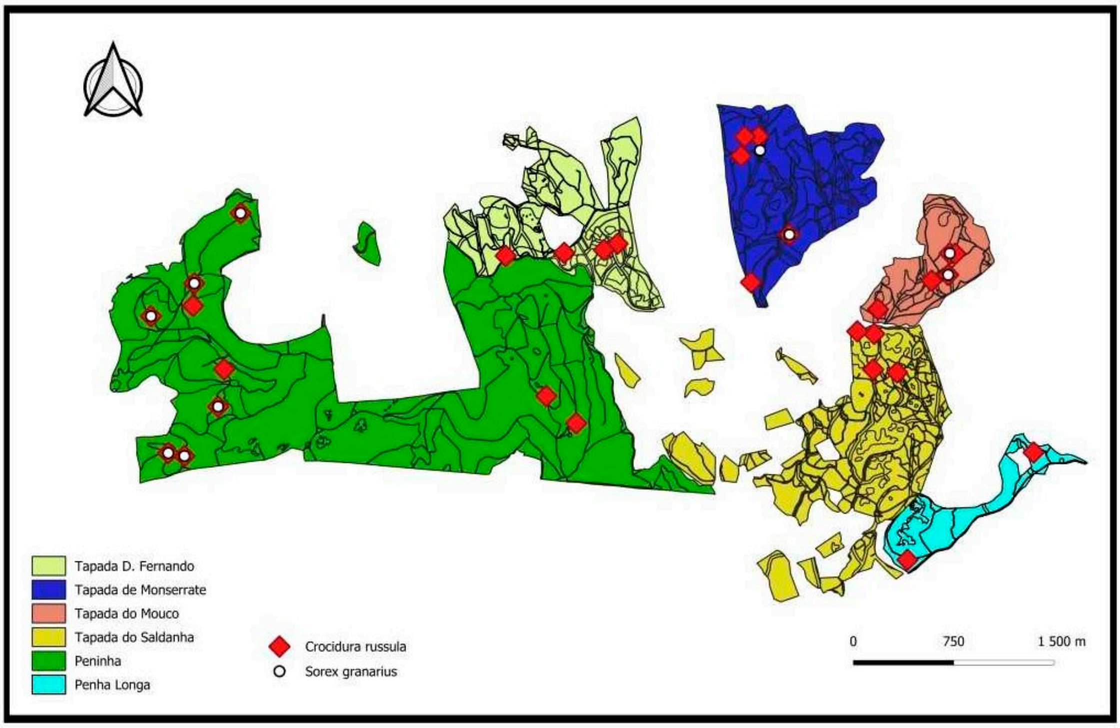

Suppl. material S5.2. Occurrence of *Crocidura russula* and *Sorex granarius* (family Soricidae) over six forested locations in the Sintra-Cascais Natural Park; species presences correspond to sites surveyed in each location as indicated in Suppl. material S1 and Figure 1 in the main text.

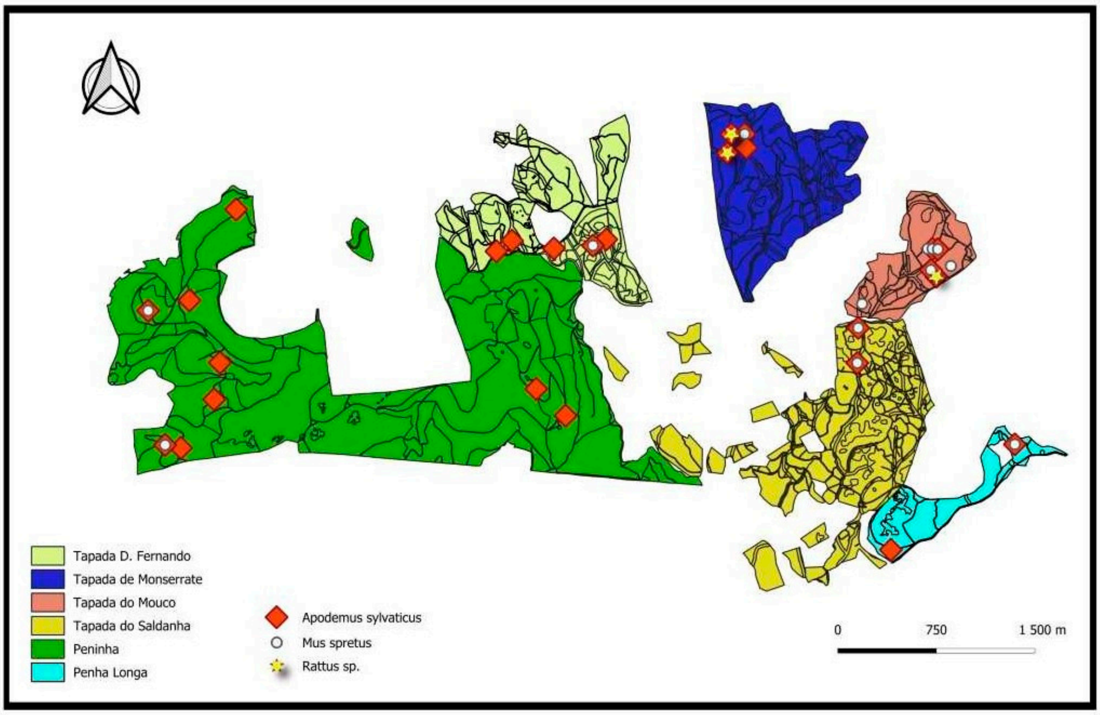

Suppl. material S5.3. Occurrence of *Apodemus sylvaticus, Mus spretus* and *Rattus* sp. (family Muridae) over six forest stands in the Sintra-Cascais Natural Park; species presences correspond to sites surveyed in each location as indicated in Suppl. material S1 and Figure 1 in the main text.

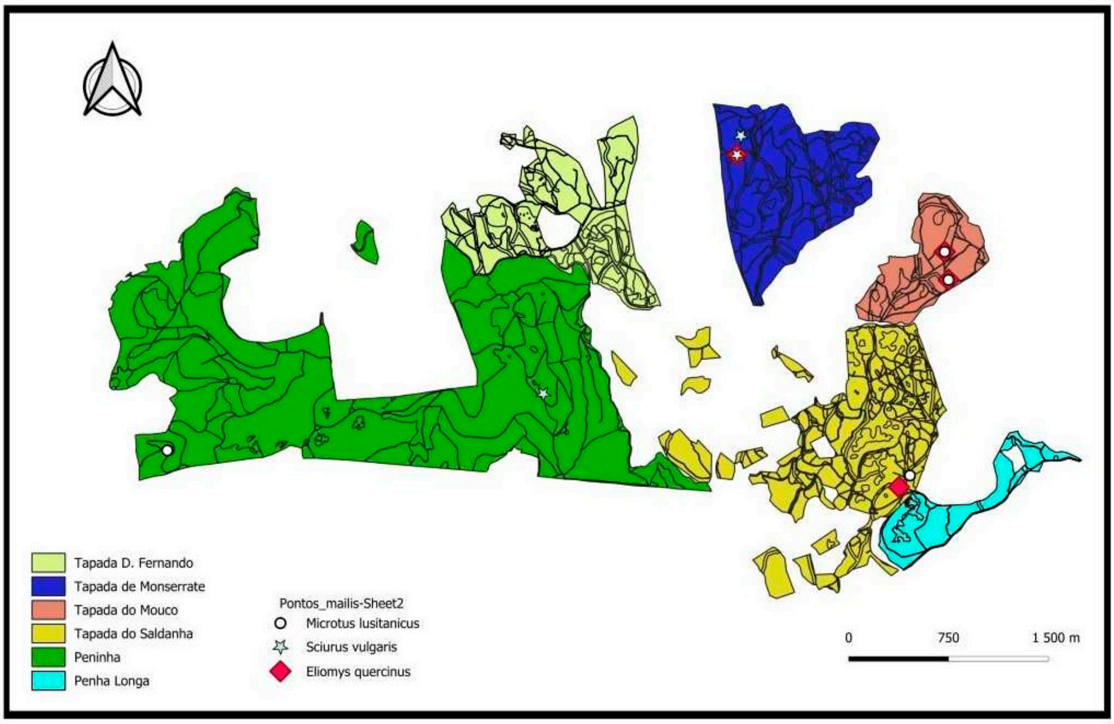

Suppl. material S5.4. Occurrence of *Eliomys quercinus* (family Gliridae), *Microtus lusitanicus* (family Cricetidae) and *Sciurus vulgaris* (family Sciuridae) over six forest stands in the Sintra-Cascais Natural Park; species presences correspond to sites surveyed in each location as indicated in Suppl. material S1 and Figure 1 in the main text.
